# Synthesis and Characterization of Bio-Composite Based on Urea–Formaldehyde Resin and Hydrochar: Inherent Thermal Stability and Decomposition Kinetics

**DOI:** 10.3390/polym17101375

**Published:** 2025-05-16

**Authors:** Bojan Janković, Vladimir Dodevski, Marija Janković, Marija Milenković, Suzana Samaržija-Jovanović, Vojislav Jovanović, Milena Marinović-Cincović

**Affiliations:** 1 “Vinča” Institute of Nuclear Sciences—National Institute of the Republic of Serbia, University of Belgrade, Mike Petrovića Alasa 12-14, P.O. Box 522, 11001 Belgrade, Serbia; bojan.jankovic@vinca.rs (B.J.); marijam@vinca.rs (M.J.); marija.kojic@vinca.rs (M.M.); milena@vinca.rs (M.M.-C.); 2 Department of Chemistry, Faculty of Sciences and Mathematics, University of Priština in Kosovska Mitrovica, 38220 Kosovska Mitrovica, Serbia; suzana.samarzija@pr.ac.rs (S.S.-J.); vojislav.jovanovic@pr.ac.rs (V.J.)

**Keywords:** polymer bio-composite, thermal stability, kinetic analysis, autocatalysis, five-membered aromatic ring compounds, graphene oxide (GO), thermal reduction

## Abstract

This work reports a study on the structural characterization, evaluation of thermal stability, and non-isothermal decomposition kinetics of urea–formaldehyde (UF) resin modified with hydrochar (obtained by the hydrothermal carbonization of spent mushroom substrate (SMS)) (UF-HC). The structural characterization of UF-HC, performed by scanning electron microscopy (SEM), Fourier transform infrared (FTIR), and X-ray diffraction analyses, showed that UF-HC consists of a large number of spheroidal particles, which are joined, thus forming clusters. It constitutes agglomerates, which are composed of crystals that have curved plate-like forms, including crystalline UF structure and graphite lattices with an oxidized face (graphene oxide, GO). The measurement of inherent thermal stability and non-isothermal decomposition kinetic analysis was carried out using simultaneous thermogravimetric–differential thermal analyses (TGA-DTA) at various heating rates. Parameters that are obtained from thermal stability assessment have indicated the significant thermal stability of UF-HC. Substantial variation in activation energy and the pre-exponential factor with the advancement of decomposition process verifies the multi-step reaction pathway. The decomposition process takes place through three independent single-step reactions and one consecutive reactions step. The consecutive stage represents a path to the industrial production of valuable heterocyclic organic compounds (furan) and N-heterocyclic compounds (pyrroles), building a green-protocol trail. It was found that a high heating rate stimulates a high production of furan from cellulose degradation via the ring opening step, while a low heating rate favors the production of urea compounds (methylolurea hemiformal (HFn)) by means of methylene ether bridges breaking.

## 1. Introduction

Amino resins include a group of products (e.g., urea–formaldehyde (UF) resins, melamine–formaldehyde (MF) resins, and benzo–guanine (BG) resins) that are used in the industry of coatings, adhesives, paper, textiles, etc. In the coating industry, amino resins are mainly used for thermoset topcoats. They produce rigid films by themselves, and when combined with the other resins (e.g., alkyd, polyester, and acrylic), they produce coatings characterized by high hardness, good chemical resistance, and good color retention [1]. The addition of amino resins to other resins can shorten the time required for coating drying, so amino resins are also classified as cross-linking agents (e.g., added to water-soluble coatings). Based on this, they are classified as slow, medium and fast cross-linking amino resins. Slow cross-linking amino resins are “butylated” and are more soluble than fast cross-linking resins, which are less “butylated”. A lower degree of alkylation increases the self-polymerization tendency of amino resins. In practice, two basic types of amino resins are mostly used: urea–formaldehyde (UF) and melamine–formaldehyde (MF) resins. The main difference between these resins is that urea–formaldehyde (UF) is made using the urea (U) and formaldehyde (F) monomers, whereas melamine–formaldehyde (MF) is made from the combination of melamine (M) and formaldehyde (F) monomers [2,3]. Both resins represent thermosetting polymers, which are obtained through irreversibly hardening (also known as the curing process) a soft solid or a viscous liquid pre-polymer.

When considering the chemical structure of UF, it contains the entity of the ‘formula’ [(O)CNHCH_2_NH]*_n_* as the repeating unit. Usually, this resin occurs as chain polymers, but it depends on the polymerization conditions. So, it can exist as a UF branched polymer or de-branched UF polymer. The conventional method of manufacturing UF resins comprises pre-condensing the urea with formaldehyde in aqueous solution under reflux at pH 7–9, and heating the mixture at pH 5–6, until the desired degree of water insolubility or viscosity is reached [4]. The UF resins are the most important type of adhesive resins for the production of wood-based panels [5]. Other than their excellent adhesive properties, they also are good electrical and thermal insulators, with good elasticity and inertness to the chemicals; so, they are used for producing sockets, different cases, and handles, and for coating electrical appliances [6]. Despite their wide use and a lot of benefits (they are colorless, have a good performance, and are fast curing and inexpensive), a serious problem related to the UF resins is the formaldehyde emission (FE). That is why it is very important to add fillers that bind free and liberated formaldehyde [7]. Practically, the low formaldehyde (F)/urea (U) mole ratio (at about F/U~1.50) represents a key parameter for lower FE [4]. The low F/U mole ratio of UF resins is necessitated by low functionality of U (≤2.30), which also limits the extent of cross-linking in the curing of UF polymers [4]. In addition, FE decreases as the mole ratio falls, but unfortunately, the other physical and mechanical properties were influenced negatively at the same time [8]. However, extensive work has reported that properties of UF resins are significantly influenced by the F/U mole ratio, and it is often done by the incorporation of excess urea, during the condensation reaction step [9]. The current procedure may remove the most of the free formaldehyde species and hemi-formals, when the hot pressing stage takes place [5].

Polymer composites are fabricated by incorporating fillers into a polymer matrix. The intent for the addition of fillers is to improve the physical, mechanical, chemical, and rheological properties of the composite [10]. Polymer composites have been used for various purposes, ranging from low-cost household products to high-performance industrial products. Despite their diverse properties, polymers have some drawbacks associated with their components, such as a low thermal stability, low conductivity, and low flame retardant properties. There are strong tendencies to resolve these issues, and one of the most effective solutions is the addition of filler in order to improve and modify the composite characteristics. Since there is a great interest in low-cost, sustainable, and environmentally friendly materials, biochar has received great interest for use as a filler, as an alternative to other non-environmentally and non-economically viable carbon fillers, such as carbon black, carbon nanotubes, and graphene [11]. Biochar is conventionally and widely used for soil amendment, or as an adsorbent for water treatment. Nevertheless, the need for transitioning to renewable materials has resulted in an expansion of biochar, for use as a filler for polymer composites [11,12]. Biochar, as a porous carbonaceous solid residue, can be obtained by the slow pyrolysis (carbonization) of biomass, at higher temperatures, ranging between T = 500 °C and T = 700 °C [13]. Similar to other carbon fillers, it is characterized by a greater thermal stability, there is a larger specific surface area, and it consists of several functional groups, such as hydroxyl, carboxyl, carbonyl, and others. The major aim of reinforcing biochar is to enhance the mechanical, thermal, and electrical conductivity properties of polymer composites [14]. Biochar can be used as a filler material in thermosets, thermoplastic, and ceramic polymer composites to improve their mechanical, thermal, and electrical properties [15]. Discussions related to properties and applications of biochar-based polymer composites were also provided by Bartoli et al. and Das et al. [16,17]. So, there are studies related to biochar as an effective filler on the properties of carbon fiber-reinforced bio-epoxy composites [18], biochar as a reinforcement filler for the styrene–butadiene rubber composites [19], the biochar reinforcing of PLA (polylactic acid) composite for fused deposition modelling [20], biochar as a sustainable and renewable additive for the production of poly(ε-caprolactone) composites [21], and many others. As for the usage of UF adhesives in composite materials, there are several important studies related to this topic, such as research described in the noted references [22,23,24]. These studies, performed by different authors, highlight the diverse approaches to improving the performance of UF adhesives in composite materials productions. The development of new materials based on renewable natural resources is a rapidly growing field of research due to the increasing demand for sustainable and eco-friendly products. This includes the use of bio-composites, based on UF resin and plant-derived (biomass) carbon materials, such as biochars. Therefore, the replacement of natural fiber with biochars to prepare bio-composites has attracted widespread attention recently. One of the breakthrough points represents the usage of hydrochar instead of bio-char. Namely, compared to slow pyrolysis, the hydrothermal carbonization (HTC) process is considered as a promising thermo-chemical conversion technology for the production of carbon-rich material (hydrochar), due to the elimination of the drying step. HTC is mostly considered economically viable for wet biomass feedstock [25]. Hydrothermal carbonization (HC) is usually carried out at temperatures ranging from T = 180 °C to T = 240 °C for *t* = 5–240 min, under sub-critical water pressures [26]. Hydrochar and biochar show different physicochemical properties, which significantly affect their potential applications. They reveal different chemical compositions and porous characteristics, as the biomass feedstock undergoes complex chemical reactions, such as degradation, dehydration, and re-polymerization in different reaction conditions (e.g., temperature, heating rate, time, and pressure). The process temperature has a significant impact on the physicochemical properties and the yield of biochar and hydrochar, as the reaction temperature influences which reaction mechanism dominates. Since hydrochar is produced via HTC in water media, the inorganic compositions of the biomass are demineralized, resulting in the reduction of ash content, and showing high affinity for both the polar and non-polar functional groups. Thus, compared to the biochar, which is produced through pyrolysis, hydrochar contains less ash content [27]. Hydrochar compared to biochar is slightly acidic, as the hydrochar contains more oxygenated functional groups. During HTC, some inorganics would be washed away in the water media, resulting in the acidic pH levels of hydrochar [28]. Generally, the literature [29] suggests that hydrochar is a valuable resource, and it is superior to biochar in certain ways, for example, it contains a reduced alkali and alkaline earth and heavy metal content, and possesses a higher heating value (HHV) compared to the biochar, produced at the same operating process temperature. Likewise, the hydrochar prepared by HTC represents an environment-friendly material, and thus, it does not generate any hazardous chemical or by-product, as other char’s products can. In the previous scientific literature, one can find papers related to the synthesis and characterization of UF eco-friendly composite material based on natural fibers [30], and the comparative study of bio-composites based on hydrochar and chitosan-modified urea–formaldehyde resins [6], but there are no detailed studies on the assessment of thermal stability properties, as well as an accurate pyrolysis (decomposition) reaction mechanism scheme of the synthesized urea–formaldehyde (UF)–hydrochar (HC) composite. Accordingly, UF bio-composite may have the potential to be a good adhesive material. Also, it should be emphasized that the use of the spent mushroom substrate (SMS) has proven to be a promising biomass precursor for hydrochar production as bio-filler, throughout hydrothermal carbonization (HTC), contributing to sustainable and economical waste management [31].

The main goal of this paper is to examine in detail the thermal stability properties of the synthesized UF resin bio-composite, where it was used as bio-filler, and the hydrochar (HC), produced from the hydrothermal carbonization (HTC) process of the spent mushroom substrate (SMS), as the starting biomass feedstock. The thermal stability characteristics were examined during the non-isothermal decomposition of the UF-HC composite at various heating rates, in an inert (Ar-argon) atmosphere. The inherent thermal properties were investigated under non-isothermal decomposition conditions for the studied bio-composite (based on the analysis of specific reaction temperatures, and characterization parameters such as the heat-resistance index (HRI), the comprehensive performance index (CPI), and integral procedure decomposition temperature (IPDT). For the kinetic analysis of the decomposition process, the model-free (isoconversional) methods and model-based method were used [32,33]. The first group of methods was used to identify and assess the degree of kinetic complexity of the investigated process. The second kinetic method was used to obtain the most reliable combination of different reaction models, which best describes the complex thermo-intensified process of bio-composites, but from a mechanistic point of view. The evaluation of the fit of the best model/method to the experimental data was carried out through rigorous statistical analysis (by using the adjusted R-squared (R^2^, a modified version of correlation coefficient, R) test, sum of squared deviations (S^2^), mean residual (MR), Student’s 95% confidence level test, and statistical F-test). The relationship between the kinetic parameters and reaction mechanisms of individual reaction components in polymer material, merged with general parameters of thermal stability, is also established. In addition to a detailed kinetic analysis of UF-HC decomposition (using the data from thermo-analytical measurements, such as simultaneous thermogravimetric analysis (TGA)–differential thermal analysis (DTA)), the physicochemical characterization of the synthesized bio-composite was carried out, using FTIR (Fourier transform infrared) spectroscopy (structural characterization) and SEM (scanning electron microscopy) (morphological characterization) techniques. Additionally, the XRD (X-ray Diffraction) analysis was used to investigate the structures of the tested sample, and for better understanding the synthesis from urea–formaldehyde (UF) resin and SMS-HTC hydrochar (sample label: UF-HC). To the best of the authors’ knowledge, this is the first study that evaluates inherent thermal stability properties during the UF-HC decomposition process, as well as the first investigation which gives a detailed insight into the reaction mechanism, that opens the channels for the production of important five-membered heterocyclic chemical compounds.

## 2. Materials and Methods

### 2.1. Materials

Urea supplied from Alkaloid (Skopje, North Macedonia) and 35% formaldehyde supplied from Unis (Goražde, Bosnia and Herzegovina) were used for the synthesis of UF resin. The chemicals used in this work, such as urea and formaldehyde, were of p.a. (pro analysis) purity, which means they meet high analytical standards and contain minimal impurities. This level of purity ensures consistent chemical reactivity, enhances the quality and performance of the final resin product, and reduces the likelihood of unwanted side reactions during synthesis procedure. Hydrochar was used as a natural bio-filler. The hydrochar was obtained from the biomass feedstock, i.e., the spent mushroom substrate (SMS) (received from the local mushroom production “Ekofungi”, located in Padinska Skela near Belgrade, the capital city) (the main ingredients of SMS are wheat straw, horse manure, and gypsum) by the hydrothermal carbonization process at temperature of T = 180 °C, under autogenous pressure, and with water medium in an autoclave reactor [31].

### 2.2. Synthesis of UF Bio-Composite

**Figure 1:**
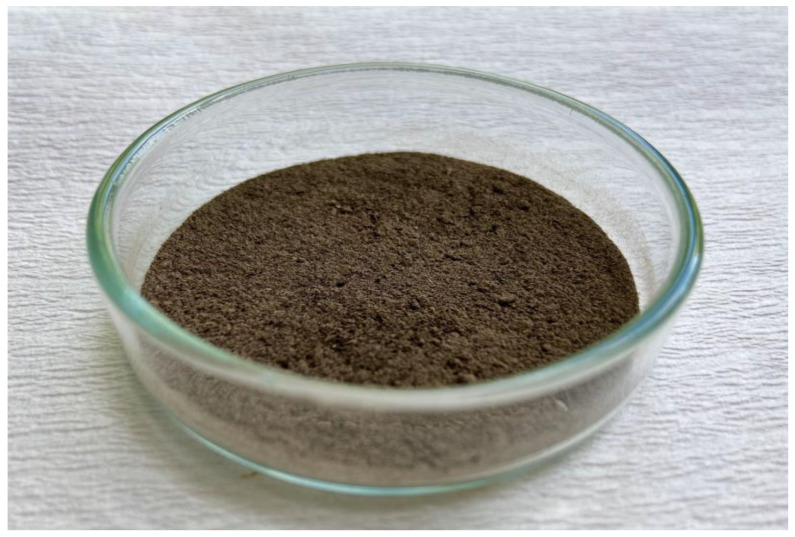
UF bio-composite (UF-HC) sample which was examined in this study.

The synthesis of urea–formaldehyde resin with hydrochar (UF-HC) was performed according to the procedure described in previous work [34]. The molar ratio of formaldehyde (F) to urea (U) (F/U) in modified UF resin was 0.80. It has been confirmed [35] that the lower the ratio of F/U, the lower the Brinell hardness, pointing to the less rigid network structure in the low F/U mole ratio of UF resin. The average particle size of the UF-HC sample was 2.32 μm (the particle size measurements were conducted using SEM photographs analyzed with ImageJ software (Developer Michael Mateas and Andrew Stern) (version 1.54k adapted for Windows platforms and the last update has been done at November 11, 2024), and the average diameter was calculated by plotting a histogram in the Origin Pro 2017 software (Pro, 2017, version 9.4) (OriginLab Corporation, One Roundhouse Plaza, Suite 303 Northampton, MA 01060, USA) and Gaussian distribution function). The external appearance of the synthesized UF bio-composite (UF-HC) is shown in Figure 1.

### 2.3. Characterization Experimental Techniques for the Synthesized UF Bio-Composite

The FTIR (Fourier transform infrared) spectroscopy, XRD (X-ray diffraction) analysis, and SEM (scanning electron microscopy) technique were incorporated to characterize the UF bio-composite in this study. The knowledge of structural properties and surface morphology, are central for the proper use of the synthesized bio-composite material.

#### 2.3.1. FTIR Analysis

The FTIR transmittance spectrum was obtained with a Thermo Nicolet 380 FT-IR (Fourier transform infra-red) spectrometer with Smart Orbit ATR (attenuated total reflectance) (Nicolet Instrument Corporation, Waltham, MA, USA). Imaging was performed in the wavenumber range from 4000 cm^−^^1^ to 400 cm^−^^1^, with the resolution of 4 cm^−^^1^ and 64 scans per spectrum.

#### 2.3.2. XRD Analysis

The XRD measurement of the UF-HC powder sample was performed using a Rigaku MiniFlex600 (Rigaku Holdings Corporation, Tokyo, Japan) diffractometer with an X-ray lamp working on 40 kV/30 mA, and the radiation source Cu Kα with a wavelength of λ = 0.15418 nm. The diffraction data were recorded in a 2*θ* range from 2*θ* = 10° to 2*θ* = 80°, counting of 10° per minute, with 0.01° steps. The crystallinity index (*CrI*) was calculated based on the XRD peak height method, using the following equation [36]:

(1) $$CrI=\frac{I_{002}-I_{am}}{I_{002}}\times 100,$$

where *I*_002_ refers to the maximum intensity of the peak, corresponding to the plane having the Miller indices 002 (2*θ* ≈ 23°), while *I_am_* represents the minimal intensity of the diffraction of the amorphous phase at 2*θ* ≈ 15°.

#### 2.3.3. SEM Analysis

The morphology of the prepared UF-HC sample was examined by the Tescan FE-SEM Mira 3 XMU (TESCAN Orsay Holding, Brno, Czech Republic) scanning electron microscope at a 20 kV acceleration voltage. Prior to SEM recording, the sample was coated with a thin gold layer by using a sputter coater (Polaron SC503, Fisons Instruments, Ipswich, UK).

#### 2.3.4. Simultaneous Thermogravimetric Analysis (TGA) and Differential Thermal Analysis (DTA) for Monitoring the Thermal Stability and Decomposition Process of UF Bio-Composite

Evaluations of the thermal stability and the non-isothermal decomposition process of the UF-HC sample were monitored by the simultaneous non-isothermal (dynamic) thermogravimetric analysis (TGA) and differential thermal analysis (DTA) techniques, using a Setaram Setsys Evolution 1750 Instrument (7 Rue de l’Oratoire, 69,300 Caluire-et-Cuire, France). The experimental specimens were heated from T = 30 °C to T = 950 °C, in a gas flow rate of *φ* = 30 cm^3^/min under the pure argon (Ar) gas, at the four different heating rates of *β* = 5.1, 10.2, 15.2, and 20.2 K/min. The average mass of the samples was about 5.0 ± 0.1 mg. Along with recorded thermogravimetry (TG) curves, the DTG (derivative thermogravimetry) curves at each heating rate used are also presented (DTG curve represents the first derivative of the TG curve, expressed in %/°C or %/min on the *y*-axis). Each measurement was repeated twice to check the reproducibility of a given measurement. It has been established that the deviation between replicates was below 0.1%.

### 2.4. Assessment of Thermal Stability of UF Bio-Composite

For the estimation of the thermo-chemical performances of the UF-HC sample in an inert (Ar) atmosphere, two characterization parameters were used. The first one represents the heat-resistance index (HRI) [37], and the second one represents a comprehensive performance index (CPI) [38]. The appropriate mathematical expressions for these indices are presented by Equations (2) and (3) [39], as follows:

(2) $$HRI=0.49\cdot \left[T_{5}+0.6\cdot \left(T_{30}-T_{5}\right)\right],$$

(3) $$CPI=\frac{{DTG}_{p}\cdot {DTG}_{mean}}{T_{i}\cdot T_{p}\cdot {∆T}_{1/2}},$$

where T_5_ and T_30_ represent the temperatures at the mass loss equaling to 5% and 30%, respectively. These values are determined from TG-curves. In the following, DTG*_p_* and DTG*_mean_* are the maximum mass loss rate (the maximum on the mass change rate curve, expressed in %/min) and the average mass loss rate (the average of the mass change rate curve, expressed in %/min), respectively (these values are determined from the differential thermo-analytical curves). T*_i_* and T*_p_* are the initial devolatilization temperature, and the temperature at the DTG*_p_*. Finally, ΔT_1/2_ represents the temperature range corresponding to DTG/DTG*_p_* = 0.5, i.e., the half-peak width range. A higher *CPI* value indicates a better thermo-chemical performance of the investigated polymer material (*CPI* is expressed in %^2^·min^−2^·°C^−3^). It should be emphasized that the initial temperature of devolatilization differs from the onset temperature (T*_onset_*) in the actual process of interest. Namely, the onset temperature, T*_onset_*, is the registered temperature on the thermo-analytical curve showing the first (origin) changes in the material under testing, in the pre-pyrolytic zone. The temperature T*_i_* is connected only to the primary (main) pyrolytic (decomposition) zone, where the abundance of gaseous products comes to the fore. At the last place, the final (ending) temperature, T*_f_*, is the temperature at which visible changes in the mass of the studied sample cease, after the completion of an entire decomposition process. Consequently, the thermal stability of UF bio-composite in respect to the heating rate applied, can be evaluated by various parameters including T*_onset_*, T*_i_*, T*_p_*, and T*_f_* temperatures, as well as the *HRI* value. Although, an efficacious and physically more meaningful parameter called the integral procedure decomposition temperature (*IPDT*) is employed to estimate the exact inherent thermal stability of the investigated material. The *IPDT* correlates the volatile parts of the polymer composites, and is expressed by following equation [40]:

(4) $$IPDT=T_{i}+A^{*}\cdot K^{*}\cdot \left(T_{f}-T_{i}\right),$$

where

(5) $$A^{*}=\frac{A_{b}+A_{c}}{A_{a}+A_{b}+A_{c}},$$

(6) $$K^{*}=1+\frac{A_{c}}{A_{b}}.$$

In Equation (4), T*_i_* and T*_f_* have the meanings described above, while A* and K* represent the constants which can be calculated by Equations (5) and (6). In Equations (5) and (6), *A_a_* and *A_b_* are the areas above and below the TG-curve, respectively, while *A_c_* is the complementary area of oblong rectangle, which was previously established [40].

It should be pointed out that one of the subjects of debate related to composite materials represents the process control, attached for efficiency optimization and accordingly eventual commercialization. The answer to this task should include the investigation of polymer composites, not only their structures, but also, and especially, deeper consideration of the temperature-dependent reaction mechanisms. The kinetic analysis of thermally stimulated heterogeneous processes (such as pyrolysis/decomposition) is capable of determining the kinetic parameters (the activation energy (*E_a_*) and pre-exponential factor (*A*)) of such processes, in order to analyze the transition states, and finally, the process reaction mechanisms. Kinetic parameters are physically meaningful in controlling the process under the current consideration, as well as to predict the thermal stability of the materials, outside of the experimental range. However, the majority of the thermally activated heterogeneous processes are kinetically complex, i.e., they can consist of several elementary reaction steps. In the next section, some of the important advanced kinetic approaches are presented, for the kinetically complex decomposition process, which includes multi-step mechanisms, as for the studied UF resin hydrochar (HC)-reinforced material.

### 2.5. Methods for Determining the Kinetic Triplet of a Process and Its Optimization

The rate of the process in the condensed state is mainly a function of temperature and degree of conversion, and can be represented by Equation (7) as follows:

(7) $$\frac{d\alpha }{dt}=A\cdot exp\left(-\frac{E_{a}}{RT}\right)\cdot f\left(\alpha \right),$$

where dα/d*t* is the conversion rate of thermal decomposition, *A* is the pre-exponential factor (1/s), *E_a_* is the apparent (effective) activation energy (J/mol), *f*(α) represents the reaction (kinetic) model or reaction mechanism function, *R* is the universal gas constant (8.314 J·(1/K)·(1/mol)), and *T* is the absolute temperature (in K). The degree of conversion (or the conversion) (α) can be obtained from the mass ratios at a given temperature or time, expressed as α = (*m*_o_ − *m_T_*)/(*m_o_* − *m_f_*) (the dimensionless quantity), where *m_o_* is the initial mass, *m_T_* is the mass obtained at the estimated temperature (or time), and *m_f_* is the final mass at analyzed instant. Equation (7) represents the origin background of the first assumption of model-free (isoconversional) kinetic analysis [41], which was based on the dependence of *E_a_* and *A* on the reaction progress (conversion, α). The second assumption is related to the fact that the reaction rate at a constant conversion value can be described as a function, which is only dependent on the temperature, *T*. Isoconversional kinetic methods are employed to examine the variation in the apparent activation energy with the degree of conversion, and therefore, the nature and complexity of the process. A condensed phase process is fairly approximated as the single-step, if the variation in its apparent activation energy with α is insubstantial; otherwise, the reaction is deemed as the following a complex reaction pathway. This kinetic approach represents a “model-free” approach, because *E_a_* is calculated independently from the reaction model (*f*(α)), so for the calculation of *E_a_*, no assumptions are made. Isoconversional kinetic methods can be isothermal/non-isothermal, differential/integral, and linear/non-linear (advanced) methods [42].

Taking the logarithm of Equation (7) gives the following linear differential isoconversional (model-free) method, known as the Friedman’s (FR) method [43]:

(8) $$ln{\left(\frac{d\alpha }{dt}\right)}_{\alpha ,\beta }=ln\left[A_{\alpha }\cdot f\left(\alpha \right)\right]-\frac{E_{a,\alpha }}{RT_{\alpha ,\beta }}.$$

where *E_a_*_,α_ values can be determined by plotting *ln*(dα/d*t*)_α,*β*_ against 1/*T*_α,*β*_, at the constant values of α at considered heating rate *β*, which demands numerical differentiation. Furthermore, the reaction type (*f*(α)) is usually not required to calculate the *E_a_*. However, it is not possible to determine the number of reaction steps, their contribution to the total effect, or the order in which they occur. In addition, when using derivative conversion data, this makes the differential isoconversional method prone to noise sensitivity and numerical instability, but modern software contain very effective filters for removing noise and background difficulties, thus obtaining reliable data and facilitating their interpretation.

Vyazovkin’s (VY) advanced model-free method [44] is a widely recommended integral isoconversional approach, for accurate determination of the apparent activation energies, *E_a_*’s. In this method, the apparent activation energy is then obtained for each value of α at different temperatures, *T_i_*(*t*), by minimizing the function, *Φ*(*E_a_*), as follows:

(9) $$\Phi \left(E_{a}\right)=\sum_{i=1}^{n}\sum_{j\neq i}^{n}\frac{J\left(E_{a,\alpha },T_{\alpha ,i}\right)\cdot \beta_{j}}{J\left(E_{a,\alpha },T_{\alpha ,j}\right)\cdot \beta_{i}}=min.$$

(10) $$J\left[E_{a,\alpha },T_{\alpha ,i}\left(t_{\alpha }\right)\right]=\int_{t_{\alpha -∆\alpha }}^{t_{\alpha }}exp\left(-\frac{E_{a,\alpha }}{RT_{\alpha ,i}\left(t_{\alpha }\right)}\right)dt,$$

where the current method uses the non-linear regression proposed by Senum and Yang, which makes it more accurate over a wider range of thermo-analytical data, and circumvents the inaccuracies related to the analytical approximation of the temperature integral [45]. However, its application remains limited as the mass transfer becomes limiting, at very high conversions (above α = 0.75/0.80 (=75%/80%)). In the above equations, *E_a,_*_α_ and *T*_α_ are the apparent activation energy and the temperature at conversion, α, respectively, obtained from the independent experimental runs *i* and *j*, and performed at the different heating rates, *β*’s. The integral is numerically evaluated by using the trapezoidal rule and the uniform grid spacing, which is continually decreased until a difference in the integral values is smaller than 10^−6^ between the consecutive interactions was obtained. This minimization can be carried out at the different values of α, to obtain the related apparent activation energy (*E_a_*) [46].

The numerical optimization (NM) method represents the model-free approach, using non-linear least square optimization. The numerical method searches the optimal functions such as *E_a_*(α) and *logA*(α) in order to obtain the best fit for the conversion (*T*, *t*). The numerical method is based on the results of the analytical differential Friedman’s method (it is often called the modified Friedman’s method (MFM)). Results of the Friedman’s isoconversional method (the curves *E_a_*(α) and *A*(α)) are optimized numerically, in order to achieve a better fit between the experimental and simulated thermo-analytical curves. The function for optimization is the sum of the squares of deviations between the measured value, *Conversion-experimental*_(*T*), and the calculated value, *Conversion-simulated*_(*T*). This sum is calculated over all the curves and over all the points in each observed curve, as follows:

(11) $$\Omega =\sum_{Curves}\sum_{Points}{\left[\alpha {\left(T\right)}_{i}^{calc}-\alpha {\left(T\right)}_{i}^{exp}\right]}^{2},$$

where α(*T*)*_i_^calc^* and α(*T*)*_i_^exp^* represent the calculated and experimental conversion values, for the considered *i*-th heating rate used. The numerical method searches the numerical values of *E_a_*(α) and *logA*(α), which minimize the function, *Ω*. Internally, each point of curves *E_a_*(α) and *A*(α) is the subject of the small changes, and for each change, the sum of squares of residuals is checked: is it better or worse than before? If better, then the new point in *E_a_*(α) or *A*(α) is saved. The iterations are repeated until no numerical improvements happen. The advantage of the numerical optimization method is reflected in the fact that it can be applied to the multiple-step reactions with the evaluation of each reaction point at various heating rates.

For all the presented conversion-dependent methods, Kinetics Neo (The NETZSCH Group Holding, Selb, Germany) computational kinetics software (Version 2.7.0.11; Build date: 21 January 2024) was used. This software, for the application of the Friedman (FR) isoconversional method, instead of the “*ln*” scale, uses “*log*” scale data, where software normally operates. Considering all the model-free methods, the kinetic parameters are determined using the points at the same conversion, between α = 0.01 (α = 1%) and α = 0.99 (α = 99%), with the conversion step increment of Δα = 0.01, from the measurements at four different heating rates (5.1, 10.2, 15.2, and 20.2 K/min). It should be noted that for the calculation of the pre-exponential factor, this software uses one of two possible approaches to find a logarithm of the pre-exponential factor (*logA*): (a) *logA* can be estimated from the intercept of the Equation (8) (FR method) and the following mathematical relations for other two isoconversional methods, on the similar principle as for the determination of *E_a_*, for known or assumed *f*(α) (usually first-order kinetics), and (b) *logA* can be found from the application of the kinetic compensation effect (KCE) [47]. Software uses the first approach, assuming the first-order reactions, in the form of the *f*(α) function, as follows: *f*(α) = (1 − α).

Model-based kinetic analysis represents the procedure for complex chemical processes, consisting of individual reaction steps, where each step can be individually connected to another reaction step (consecutive, competitive, independent, etc.), in order to build a kinetic model of the complex process under study. The model-based kinetic approach describes the reaction rate of multi-step chemical reactions by the system (or the set) of kinetic equations, where each reaction step has its own kinetic equation and own kinetic triplet, containing the activation energy (*E*), the pre-exponential factor (*A*), as well as the reaction type function (*f*(α)), as shown in Table 1. Kinetics Neo uses the multivariate non-linear regression method (MVarNLRM) to resolve concentration equations in a multi-step process.

Namely, the model-based approach allows the determination of the reaction mechanism by the minimization of differences between experimental and calculated values. Accordingly, the function *f*(α) is used to describe the rate limiting the mechanistic reaction, which is chosen from a row of tabulated functions (Table 1), based on the data obtained from a preliminary best-selected model-free estimation. Therefore, the result of the model-based kinetic analysis provides information about the reaction mechanism, the form of equations for the elementary reaction steps, and the values of kinetic triplets [i.e., *E*, *A,* and *f*(α)].

In this paper, a complete model-based kinetic analysis was carried out using Kinetics Neo software (The NETZSCH Group Holding, Selb, Germany) (Version 2.7.0.11; Build date: 21 January 2024), and it was based on models that include several process steps, in which the individual steps can be linked as independent, parallel, competing, etc. For each of the models selected, the reaction type for each step has some unknown kinetic parameters, such as the activation energy (*E*), the pre-exponential factor (*A*), and the reaction order, as well as the contribution of each step to the entire process. All the unknown parameters can be found from the fit of measured data, with simulated thermo-analytical curves. The statistical comparison of the fit for different models allows one to select an appropriate model, with a corresponding set of kinetic parameters [49].

Related to the kinetic parameters estimation, the kinetic compensation shows a strong positive correlation between the effective activation energy (*E_a_*) and the pre-exponential factor (*A*), for a reaction between the same reactants under similar experimental conditions, or similar reactants under the same conditions, even though these parameters are supposed to be independent [50]. According to the insightful papers [51,52], the kinetic compensation effect (KCE) was based on the mathematical source of the linear relationship, as follows:

(12) $$logA_{\alpha }=a+b\cdot E_{a,\alpha },$$

where *a* and *b* are the constant coefficients, for a series of related rate processes. Namely, the KCE means that the alteration in *E_a_* values will prompt a complementary compensating response in *A*, which can also be used to test the experimental results. The coefficients *a* and *b* represent the intercept and the slope of the regression line, equal to *logk_iso_* = *a* (*k_iso_* = 10*^a^*) and 1/*RT_iso_* = *b*, where *T_iso_* = 1/*R*·*b*. The *k_iso_* and *T_iso_* are the iso-kinetic rate constant and iso-kinetic temperature, respectively, where at the temperature *T_iso_*, all the chemical compounds studied are characterized by the same rate constant, equal to the *k_iso_*. The difference in decomposition mode for solid-state reactions is the most common cause for the appearance of KCE. KCE in fact provides a possible means to predict the effects of experimental factors on kinetic parameters. According to this relationship, for any change in the experimental *E_a_*, arising from variation in experimental conditions, a corresponding change in the pre-exponential factor also occurs; thus, we could correlate different parameters under different experimental conditions. The true KCE can prove to be useful in chemical research, for identifying the governing reaction mechanism in the process under investigation.

The linear correlation between *logA* and *E_a_* can be expressed through Equation (13), including rigorous physicochemical meaning related to intrinsic kinetic parameters (*A_int_*, *E_a_*_,*int*_) and thermodynamic parameters (Δ*_r_H*°—the change of the standard reaction enthalpy (J/mol), and Δ*_r_S*°—the change of the standard reaction entropy (J(K·mol)^−1^)), as follows:

(13) $$logA=\left(logA_{int}-\frac{{∆}_{r}S^{o}}{R\cdot {∆}_{r}H^{o}}\cdot E_{a,int}\right)+\frac{{∆}_{r}S^{o}}{R\cdot {∆}_{r}H^{o}}\cdot E_{a}.$$

The KCE is often correlated with the concept of the isokinetic point (IKP). The IKP refers to a common point of intersection of the Arrhenius lines. Comparing with Equation (12), it can be observed that for all rate processes, whose kinetic parameters are in the parameter set which satisfies Equation (12), the corresponding Arrhenius plots have a common point of intersection [*logk_iso_*, *T_iso_*^−1^], so the Equation (12) can be re-written as follows:

(14) $$logA_{\alpha }=logk_{iso}+\frac{1}{R\cdot T_{iso}}\cdot E_{a,\alpha }.$$

In the case of the isoconversional methods, the IKP that appeared at different conversion (α) values varied as the reaction progresses. So, according to Equation (13), the quantity *T_iso_* can be expressed as follows:

(15) $$T_{iso}=\frac{{∆}_{r}H^{o}}{{∆}_{r}S^{o}}\;\left[K\right].$$

The following equation shows the linear relationship for thermodynamic equilibrium, as follows:

(16) $${∆}_{r}H^{o}=\underset{intercept}{\underbrace{{∆}_{r}G_{T_{iso}}^{o}}}+\underset{slope}{\underbrace{B}}\cdot \;{∆}_{r}S^{o},$$

where the quantity *B* has the dimension of temperature (in Kelvins, K), and it is often defined as iso-equilibrium temperature (*T_eq_*) and the corresponding behavior is called the iso-kinetic or iso-equilibrium effect, because it seems that at the temperature *T_eq_*, all the reactions in the series should have the same rate (or equilibrium) constant [53]. The intercept in the linear correlation between Δ*_r_H*° and Δ*_r_S*°, expressed through Equation (16), represents the standard Gibbs free energy reaction change, at a specific iso-equilibrium temperature. When *T* = *T_eq_*, Δ*_r_G*° becomes the same for all reactants, *T_eq_* represents the temperature at which Δ*_r_H*° and Δ*_r_S*° are completely compensated. It is an axiom of extra-thermodynamic relationships, that all sets of reactions which exhibit enthalpy-entropy compensation are governed by a single mechanism, and all related reactions, which have the same compensation temperature, take place via the same reaction mechanism [54]. As for kinetic compensation, when thermodynamic compensation occurs, the mechanism is the same for the entire range of the experimental variables, which are covered substantially.

## 3. Results and Discussion

### 3.1. FTIR Results

**Figure 2:**
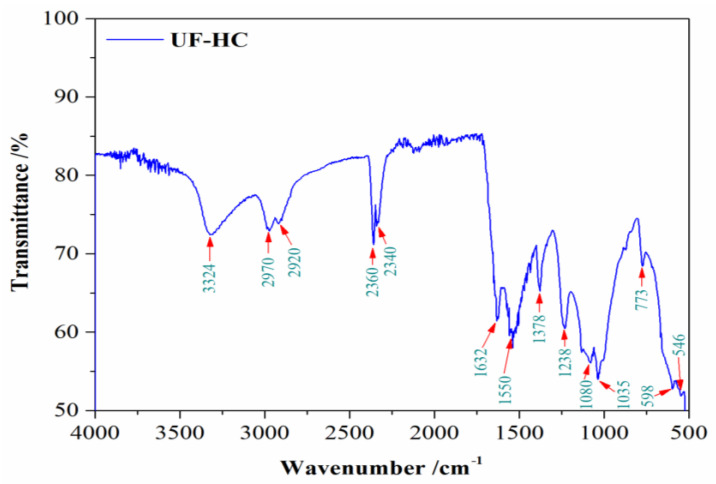
FTIR-ATR spectrum of powdered UF bio-composite (UF-HC), with the marked specific vibrational bands (expressed in cm^−1^).

FTIR spectroscopy in the attenuated total reflectance (ATR) mode represents a non-destructive and essential characterization technique to elucidate the structure of tested specimen at molecular scale. Thus, this technique permits the determination of components or groups of atoms that absorb in infrared at specific frequencies, permitting the identification of the molecular structure. The FTIR-ATR spectrum of UF-HC is shown in Figure 2.

The resulting FTIR-ATR spectrum is characteristic for the synthesized bio-composite sample with clearly separated regions related to each of the phases, counting the polymer matrix and the biological filler from derived hydrochar (HC). Namely, the broad band situated at 3324 cm^−1^ can be assigned to the strong N–H stretching of secondary amides [55,56]. Urea contains primary amines, which indicates that the urea might be fully reacted during the resin preparation and curing process, so this band reflects the characteristic functional group of the UF resin. The vibrational peaks at 1632 cm^−1^ and 1550 cm^−1^ can be attributed to the stretching of carbonyl group (C=O), and C–N stretching of secondary amines, respectively [55,56]. The vibrational band, which appears at the wavenumber position of 1378 cm^−1^, is a characteristic band attached to –CH_2_OH, illustrating a typical reaction between urea (U) and formaldehyde (F) [55]. The intense peak at 1238 cm^−1^ can be attributed to the stretching of C–N and N–H of tertiary amines [55,57]. The emphasized peak at 1035 cm^−1^ is assigned to the methylene bridge (–NCH_2_N–) [55,58].

The peak positioned at 1080 cm^−1^ belongs to the aliphatic ethers (C–O deformations) [59,60]. The vibrational band at 2920 cm^−1^ is attributed to the asymmetric stretching vibrations *ν*(C–H) in the methylene (–CH_2_) group. The specific vibrational band located at 1035 cm^−1^ belongs to the C–O–C band, which represents O-containing functional groups of hydrochar (HC) [61]. The weak peak at 773 cm^−1^ belonged to the aromatic C–H out-of-plane deformation, probably due to the presence of lignin residue in HC [61,62]. The absorbance peak located at 2970 cm^−1^ represents CHn stretching vibrations, pointing to aliphatic (*al*.) and aromatics (*ar*.) [62,63]. The two vibrational peaks at positions of wavenumbers of 2340 cm^−1^ and 2360 cm^−1^ can be attributed to –OH stretch from the strong H-bonded-COOH [64]. These peaks in the indicated wavenumbers range (2340–2360 cm^−1^) could also be assigned to the CO_2_ that was absorbed during the recording of the FTIR spectrum [65]. The remaining two identified vibrational bands originate from impurities present in the UF-HC sample. The peak located at 546 cm^−1^ is attributed to coupling between the O–Si–O bending vibration and the K–O stretching vibration [66], while the peak located at 598 cm^−1^ (Figure 2) can be assigned to Si–O–Si bending vibration in depolymerized structural units of quartz [67]. All the obtained vibrational bands on the measured FTIR spectrum are autonomous and reflect an excellent agreement with the effective transfer of UF polymer matrix, and the organic filler, into the newly synthesized product—UF-HC. The FTIR results are also in very good agreement with studies reported earlier [68,69,70,71]. It should be emphasized that identified impurity in the form of quartz originates from biomass source in SMS, and it is closely related to its constituents, primarily wheat straw and gypsum [72,73].

### 3.2. XRD Results

**Figure 3:**
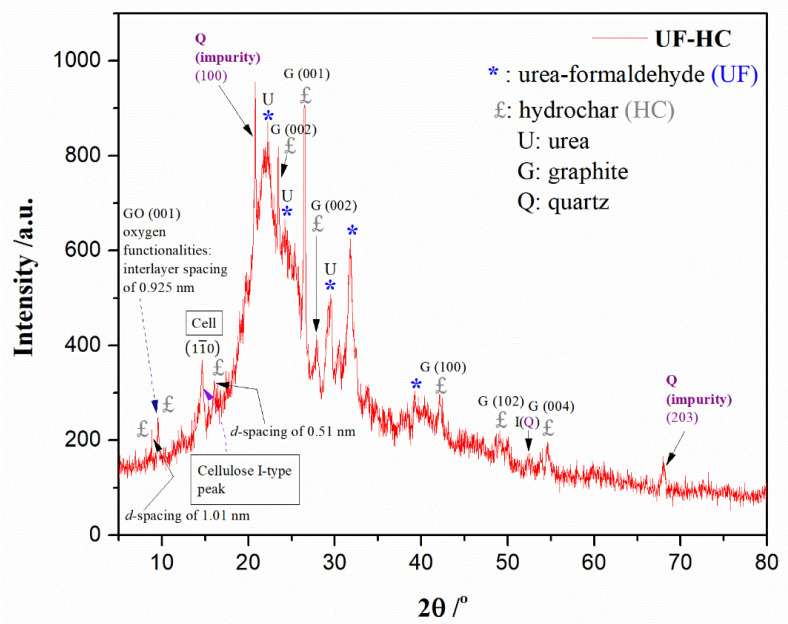
The XRD diffractogram (XRD pattern) for UF-HC sample (Abbreviation: Cell ≡ cellulose).

X-ray diffraction (XRD) is an important and widely used material characterization instrumental technique. Consequently, this technique enables obtaining information about the degree of crystallinity for semi-crystalline, amorphous polymeric, and composite materials. In that context, XRD is a very useful technique for the identification and confirmation of the preparation of the urea–formaldehyde (UF) resin/hydrochar (HC) composite material (UF-HC). Figure 3 shows the XRD pattern for the synthesized UF bio-composite (UF-HC sample).

The obtained XRD profile clearly suggests the existence of crystalline phases and amorphous regions in the UF-HC composite material. So, the sharp peaks at 2*θ* = 22.2°, and 24.3° and wide peak at 2*θ* = 31.8° are typical X-ray diffraction peaks of urea–formaldehyde (UF) [74]. The broader peak located at 2*θ*~22° is the proof of the presence of an amorphous area in the material, but the shape of the XRD peak of UF indicates increased crystallinity (it prevented the formation of the cross-linked network after curing; decreased F/U molar ratio (~0.80)).

One additional XRD peak of UF with a lower intensity appears around 2θ~40° (Figure 3), but no new peaks at higher Bragg’s angles at 2*θ* ≈ 47° and 57°, which would then be attributed to interactions between UF and SiO_2_, resulting in the formation of hydrogen bonds between UF and silanol groups of SiO_2_ [57]. However, as designated with I(Q) in Figure 3, one smaller, sharper peak at a position around 2*θ* = 52° is identified, which may indicate a possible interpenetration of SiO_2_ into the UF resin [57]. In the XRD diffractogram, one sharper diffraction peak is also observable, located at 2*θ* = 29.8°, belonging to the (003) crystal plane of urea [75].

It should be noted that UF resin with a low F/U molar ratio is less branched and more linear in structure, compared to the UF resin with a high F/U molar ratio, and this could be due to hydrogen bonds between the linear molecules thereby keeping the ordered arrangements (see discussion above). It was found [76] that UF resin with a lower F/U molar ratio exhibits two sharper peaks with greater intensities at positions of 2*θ* = 22.2° and 2*θ* = 24.3°, respectively (Figure 3). The two additional peaks that appeared at about 2*θ*~31° and 2*θ*~40° indicate supplementary crystalline regions, for the used UF resin. Therefore, in our considered case, UF resin with a lower F/U molar ratio (~0.80) shows a more crystalline structure (Figure 3) [76,77]. Within Bragg’s angles region of 2*θ* = 20–25°, a very sharp quartz peak appears at 2*θ* = 20.8° with crystal plane (100) (Figure 3) [78,79], which represents a typical impurity content present in the UF-HC sample. Also, one additional quartz peak (Q (203) plane) [79,80] appears at the position of 2*θ* = 68° (Figure 3).

From the obtained XRD pattern of the UF-HC sample, a graphite sharp peak can be observed at 2*θ* = 26.5° (Figure 3), which can be attributed to hexagonal interlayer spacing (001) [81]. In the 2*θ* region, between 2*θ* = 41° and 2*θ* = 47°, reflection (100) was identified at around 2*θ* = 43° (Figure 3), suggesting the existence of randomly stacked graphene sheets [81]. This also indicates that in the current amorphous domain, “non-graphitic” carbon may appear [82]. In addition, graphite reflection (102) with a weaker intensity at around 2*θ* = 50° [83] is also observed (*d*-spacing is 0.18 nm) (Figure 3). In addition, the reference peak for the graphite (004) at 2*θ* = 54.6° [84] was also identified. From the presented XRD profile, the diffraction peaks at 2*θ* = 23.5° and at around 2*θ* = 43° can be attached to the disordered graphitic (002) and (100) planes, respectively [85]. It should be noted that the above-indicated peak at 26.5° (interlayer distance of 0.339 nm) is the referent point that probably originates from graphene layers (preliminary already hinted—see above discussion), which makes up the carbon structure of HC in the synthesized UF-HC composite. The proposed structure is indeed supported by the appearance of the diffraction peak at 2*θ* = 27.8° (Figure 3).

However, at the shorter 2*θ* angles, there is one sharp narrow peak at about 2*θ* = 17°, corresponding to the *d*-spacing of 0.51 nm, and one even sharper, but very narrow peak at 2*θ*~8.8°, corresponding to the *d*-spacing of 1.01 nm [86] (Figure 3). This indicates that the most important spacings are not randomly determined, but may be due to the “linker” between lattices. So, the occurrence of these peaks suggest a polycrystalline sample, and also a lot of minor variation, potentially resulting in similar, but distinct structures. This is supported by the appearance of a very narrow and sharp single peak at 2*θ* = 9.5° (Figure 3), which corresponds to “oxidized” graphite, i.e., graphene oxide (GO) (graphene oxide (GO) basal plane (001)—Figure 3), and refers to the interlayer spacing of 0.925 nm [87,88]. Therefore, the synthesized UF-HC composite material contains lattices with an oxidized face, which increases the lattice spacing to account for oxygen-containing groups. Considering these results with ones obtained from FTIR measurement (Figure 2), there is excellent agreement among the results so far.

In the continuation of this discussion, it is necessary to pay special attention to the appearance of a pronounced diffraction peak at 2*θ* = 14.9° (Figure 3), which represents a characteristic peak for residual cellulosic material, i.e., cellulose I-type peak, and this can be referred to cellulose I crystallographic plane ($1\bar{1}0$) [36]. This diffraction peak is evidence of the presence of a cellulose I crystalline structure produced from hydrochar (HC). Namely, this is proof that speaks of the retention of cellulose’s own properties, and the incomplete carbonization of biomass feedstock (SMS), under mild hydrothermal conditions. Under the actual hydrothermal conditions for the thermo-chemical treatment of SMS to produce hydrochar, hemicelluloses component degrades much faster than cellulose, while lignin behavior is more inert, but its decomposition kinetics are strongly dependent on the set of conditions, during the hydrothermal processing of SMS (lignin component is fragmented and dissolves with an increase in reaction time). Obviously, for the hydrothermal carbonization process implemented here, the mentioned components in the biomass used in this study have less tight bonds than the cellulose (Cell) (Figure 3).

From XRD data, the crystallinity index (*CrI*) was calculated using the Segal equation, as previously established. The obtained value of crystallinity index was *CrI* = 54.95%. One of the reasons for the rather high *CrI* lies in a more progressive removal of amorphous non-cellulosic materials (hemicelluloses and lignin), reducing the amorphous contribution, and increasing the crystallinity of investigated sample. This is a good reason for the required feature, for using SMS-HC as bio-filler, in the development of such bio-composites. On the other hand, the crystalline region has a higher chemical stability than that of the non-crystalline region in the UF bio-composite. The increase in crystalline regions enhances the water resistance of the synthesized material. Correlated with this fact, it was also reported [89] that the crystallinity of UF resin may increase with the treatment of hydrolysis [34], considering the greater decomposition of amorphous regions. Obviously, for the manufactured UF bio-composite, the elevating of *CrI* implies that the percentage of amorphous regions in the UF-HC sample decreased (Figure 3). At the same time, it must be borne in mind that UF resin with a lower F/U molar ratio contains more linear molecules forming crystal regions by hydrogen bonds, and thus augmentative crystallinity (see also the discussion above, as well as the general appearance of the XRD spectrum).

### 3.3. SEM Results

**Figure 4:**
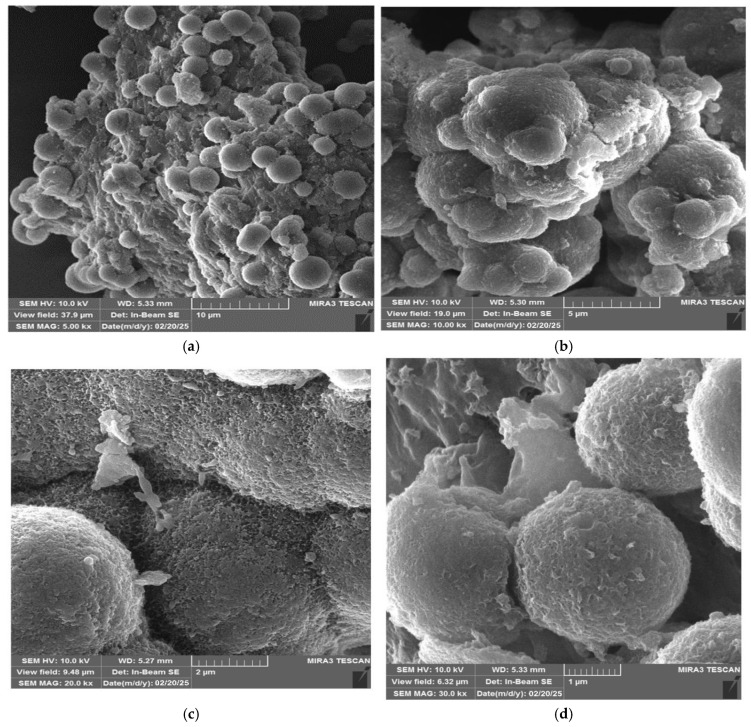
Scanning electron microscopy (SEM) images of UF-HC composite, at different magnifications: (**a**) 5.00 kx, (**b**) 10.00 kx, (**c**) 20.0 kx, and (**d**) 30.0 kx, respectively.

Figure 4 shows the morphological structure of the UF-HC sample, represented in a form of SEM images at different magnification scales: (a) 5.00 kx, (b) 10.00 kx, (c) 20.0 kx, and (d) 30.0 kx, respectively. The synthesized UF-HC specimen is characterized by a bio-polymer–UF resin microspheres structure, which retains its original morphology and uniform particle size (Figure 4a). The SEM image at a low magnification (Figure 4a) shows that UF-HC had a more spherical and rounded shape, with a small difference in the particle size.

The SEM image under the higher resolution (Figure 4b) shows that the synthesized sample still has a spherical, plump, and relatively uniform particle size distribution, but they aggregated more severely. However, the SEM images under high magnifications, such as Figure 4c,d, clearly show that the particle size is relatively small (the average diameter ranges between 2.0 µm and 2.5 µm), whereby the degree of agglomeration of microcapsules and their strength largely depends on the used F/U ratio. Scanning electron microscopy (SEM) (Figure 4d) showed that the microcapsule walls possessed a rough surface. Namely, the capsule roughness (Figure 4c,d) has been attributed to the precipitation of polymerized urea–formaldehyde from the water phase and its deposition onto the capsule wall during in situ polymerization [34]. This shell roughness is desirable as it promotes the ‘capsule’ adhesion to the polymer matrix, and provides a greater possibility for microcapsule rupture in the event of the propagation of cracks [90]. It should be noted that the particle sizes and structure of the microspheres can be changed with temperature. Namely, with the increase in the temperature [34], the particle size of produced microsphere can be significantly reduced, while the surface can change from a porous surface structure to a dense surface structure (Figure 4c). It was found that the standard deviation of microsphere size decreases continuously with an increase in the temperature, indicating that the distribution of microspheres tends to be more uniform, and this is attributed to the decomposition of urea at higher temperatures [91]. The increase in the temperature results in more decomposition of urea, leading to the decrease in the urea-to-formaldehyde ratio. So, such a decrease produces smaller-sized UF-HC microspheres.

### 3.4. TG-DTG-DTA Measurements of UF-HC Composite Decomposition

**Figure 5:**
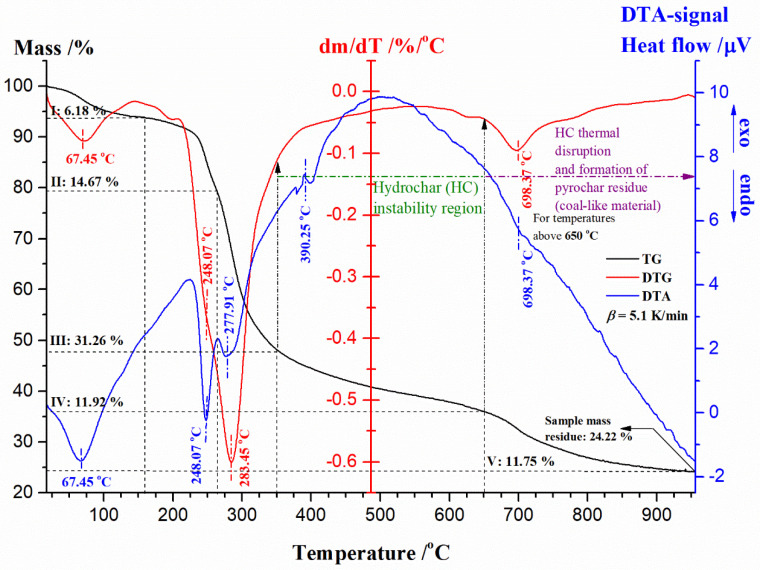
Simultaneous TG-DTG-DTA curves of the non-isothermal thermal decomposition process of UF-HC composite at *β* = 5.1 K/min. The corresponding stages in the mass loss of the sample are also marked, as well as the positions of the specific temperature values, which characterize the entire process. In the same figure, the sample mass residue was also indicated.

Figure 5 shows a simultaneous display of thermogravimetric (TG—expressed as % vs. °C), derivative thermogravimetry (DTG—expressed as %/°C vs. °C), and the differential thermal analysis (DTA—expressed as heat flow/μV vs. °C) curves, at the heating rate of *β* = 5.1 K/min, for the non-isothermal decomposition of UF-HC, in an inert (Ar) atmosphere. From the results presented in Figure 5, the first (I) sample mass loss (~6.18% with *max*. *T* at 67.45 °C, and ranges between ambient temperature and 160 °C) corresponds to the evaporation of water, formaldehyde releases, then scission weak linkages like hydrogen bonding, and particularly to the transformation of methyl ether bridges into methylene bridges [92,93], leading to formaldehyde emission. The removal of water results from a polycondensation reaction between hydroxymethyl and amine groups in a resin structure [93]. This stage is characterized by an endothermic effect. After this, the second (II) (~14.67%) and the third stages (III) (~31.26%) occur, together making the largest loss in the mass of the sample (the total ~45.93%) (Figure 5), and represent the rapid decomposition region of UF-HC. Namely, the second (II) stage is characterized by the appearance of a “shoulder” on the DTG curve, and a peak appears on the correspondent DTA curve, at the same temperature value of 248.07 °C (Figure 5). Next, the third (III) stage is reflected in the main peak on the DTG curve and DTA peak, with temperature differences at a maximum of 5.54 °C (Figure 5). Both of these stages are characterized by endothermic events, and actually determine two temperature points of UF resin decomposition in UF-HC sample, and they are 248.07 °C and 283.45 °C (considering DTG-curve) (both decomposition stages cover a temperature range from approx. 200 °C up to 350 °C). Based on the external appearance of the DTG-curve, with the “shoulder” peak manifestation, it is likely that these decomposition process steps of UF-HC have overlapping temperature behavior (it can be assumed that the temperature region, where the “shoulder” peak and the main DTG peak appears, can be directed towards the rupture of the methylene ether link related to UF resin structure—this is the main reaction area in which resin destruction takes place, where initial devolatilization temperature is transferred to the higher temperatures; therefore, it may follow that the lower quality loss causes good thermal stability (the latter largely depends on the UF synthesis procedure)).

**Figure 6:**
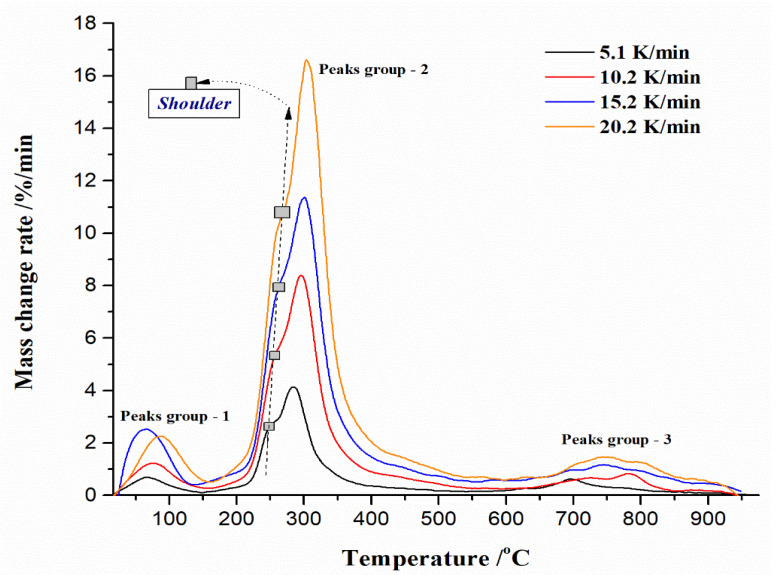
DTG curves (mass change rate (%/min) vs. temperature (°C)) of the non-isothermal thermal decomposition process of the UF-HC sample, at the different heating rates (*β* = 5.1, 10.2, 15.2, and 20.2 K/min). Corresponding groups of reaction peaks, as well as “shoulder” peaks, are also marked in the same figure (position of “shoulder” peak at different heating rates is marked by gray square).

It can be observed that the main DTG peak takes place at higher temperatures, closer to the *T* ~ 300 °C, which may suggest that the dominant reaction here could be the conversion of methyl ether functional groups into methylene functional groups, where a temperature shift occurs at higher temperatures. So, it appears to be a possible consequence of interactions between the cellulose residue and UF [92]. This effect was transferred into another decomposition stage of UF-HC (stage IV, with sample mass loss of ~11.92%, and which occurs in temperature range from 380 °C to 650 °C), where a smaller and sharper exothermic peak takes place at 390.25 °C (Figure 5), which enters into the HC instability reaction region. This may encompass the cross-linking reactions, which are responsible for char-residue formation (the formation of cross-linking structures); so, the percentage of carbon (C) increases (carbon-rich residue) [94]. Within stage IV, the decomposition of lignin and cellulosic-derived compounds can also be performed. It should be noted that in the current stage, a “secondary” char product may be produced, which arises from sugar-derived compounds dissolution in the liquid phase (such as HMF (5-hydroxymethylfurfural)) by the condensation and re-polymerization reactions [95]. In the considered temperature zone (between 380 °C to 650 °C), we can also expect decomposition reactions of oxygen-containing functional groups, such as carboxyl (–COOH) and carbonyl (C=O) groups on the surface of the HC constituent in the UF-HC sample, with CO_2_ emissions. A further increase in temperature leads to the final process stage (stage V, with sample mass loss of ~11.75%, that takes place for temperatures above *T* = 650 °C), which is characterized by a broader DTG peak and the endothermic effect at ~698.37 °C (Figure 5). This stage can be attributed to the decomposition of oxidized sp^2^ structures in the graphitic core of the UF-HC sample (with an additional formation of the pyrochar residue). Figure 6 shows DTG curves (expressed as the mass change rate curves in the %/min units) of the thermal decomposition process of the UF-HC sample, at different heating rates (*β* = 5.1, 10.2, 15.2, and 20.2 K/min) in an inert (Ar) reaction atmosphere.

The first group of peaks (“Peaks group-1” (Figure 6)) extends over the temperature range of Δ*T*_1_ = RM (the room temperature)—approx. 200 °C, and these peaks can be attached to methylene ether bridges transformation into methylene bridges, and branching and cross-linking reactions. Above 200 °C (the second group of peaks—“Peaks group-2”—(Figure 6)), the decomposition process probably involves C–N bond scission (corresponds to position of “shoulder” at about *T* ≈ 250 °C). However, with a further increase in the process temperature, competition between chain scission and cyclization may take place, forming stable chemical structure products, at about *T* ≈ 300 °C (the main DTG peak, within “Peaks group-2”) [96]. These products may suffer further temperature-intensified fragmentation above 300 °C, which would correspond to the descending part of the DTG curve (on the right wing of DTG curves at various heating rates) in the temperature range between 350 °C and 425 °C. Fragmentation products can be accumulated in the next stage of decomposition, where oxygen functionalities degrade (with temperature range between approx. 430 °C and 600 °C) (see the above discussion). With a further rise in the temperature, above 600 °C, the third group of peaks (“Peaks group-3”—(Figure 6)) takes place, and they are attributed to the decomposition of sp^2^ carbon and other organic-like structures, in the core of UF-HC. Considering actual reaction zones, the abundance of volatile products, such as H_2_O, CO_2_, CO, HCNO, HCN, and NH_3_ gases takes place [97].

### 3.5. Inherent Thermal Stability Analysis Estimated from Thermo-Analytical Measurements

Dynamic TG-DTG measurements were applied in this study for the assessment of thermal stability, for the UF-HC composite, regarding the applied heating rates (*β* = 5.1, 10.2, 15.2, and 20.2 K/min). For this purpose, the following values of characteristic parameters important for this analysis were derived: *T_onset_* temperature as the basic thermal stability parameter, the “shoulder” peak temperature, *T_sh_*, the peak (maximum) temperature, *T_p_*, the initial devolatilization temperature (*T_i_*), the final decomposition temperature (*T_f_*), *T*_5_, *T*_10_, *T*_30_, and *T*_50_ which correspond to temperatures of 5%, 10%, 30%, and 50% of the mass losses on TG-curves, at different heating rates (see Figure S1, Supplementary Material), respectively. The values of the maximum mass change rate (*R_max_*) at the *T_p_*’s were also determined. Likewise, the values of the comprehensive performance index (*CPI*), the heat-resistance index (*HRI*), and the integral procedure decomposition temperature (*IPDT*) were also established. The values of the obtained parameters for the UF-HC composite are listed in Table 2.

From the results presented in Table 2, it can be observed that all the specific reaction temperatures increase with an increasing heating rate, moving towards higher values.

As the heating rate increases, the maximum (peak) decomposition temperature (*T_p_*) is shifted from 279.49 °C at 5.1 K/min to 303.91 °C at 20.2 K/min (the *T* difference amounts to ~24.42 °C), while the maximum decomposition rate (*R_max_*) also increased from 3.420%/min at 5.1 K/min to 16.616%/min at 20.2 K/min. The thermo-analytical curves are shifted to higher temperatures, due to the heat transfer enlarging with increasing the heating rate. Also, both *T_onset_* and *T_i_* increase with an increasing heating rate, so, transformations related to these temperatures are dependent on the heating rate used, whereby a symmetric shift in their values with *β*’s takes place. The situation is similar in the case of temperatures *T*_5_, *T*_10_, *T*_30_, and *T*_50_. The heating rates do not affect the change in the shape of both the TG (Figure S1) and DTG curves (Figure 6), but the increase in *β*-value leads to their movement into higher temperature regions. The reason for this shift is probably related to differences in the heat transfer and kinetic rates, indicating that the decomposition rate of reaction products may slow down as the heating rate increases. When the heating rate increases, the heat transfer resistance may cause a larger temperature difference between the heater and the sample, thus delaying UF-HC decomposition. Furthermore, at higher heating rates, the time required to reach the decomposition temperature becomes shorter, and then the temperature difference between the tested sample inside and outside may turn to be larger as well, subsequently causing thermal lagging that may cause a delay in the thermal decomposition. This can be observed from Figure 6, since the main DTG peak becomes stronger and wider with an increasing heating rate from 5.1 K/min to 20.2 K/min, and these facts are confirmed by both promoted *T_sh_* and *T_p_* temperatures, which are shown in Table 2. The *T_f_* temperatures at all heating rates are positioned at high values, pushing the process towards the high-temperature zone (*T_f_* rises with elevating the heating rate, *β*), where HC significantly affects the thermal stability of the sample, in which a larger amount of heat energy is required to break down the sp^2^ hybridized carbon atoms. Considering *T_onset_* values, they tend to go up when the heating rate increases. These results may suggest that when the working temperature approaches *T_onset_*, a significant mass loss of the sample may occur.

**Figure 7:**
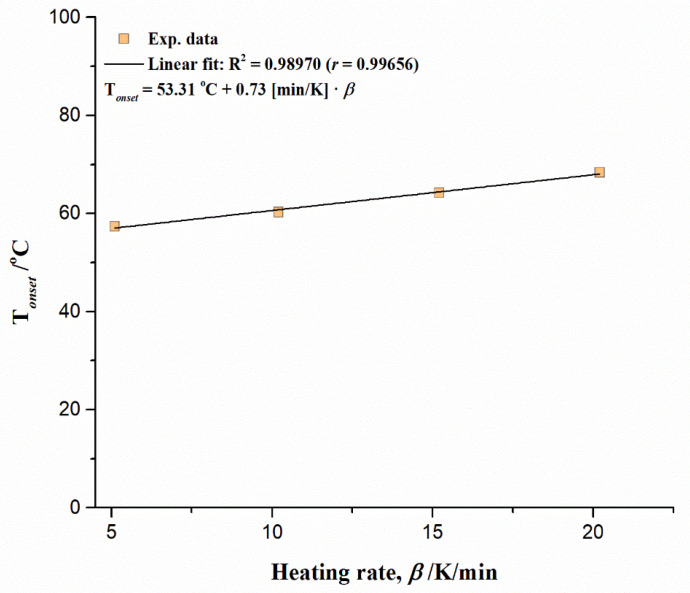
The influence of heating rate (*β*) on onset temperature (*T_onset_*), for the non-isothermal thermal decomposition process of UF-HC composite.

The influence of heating rate (*β*) on *T_onset_*, in the case of the non-isothermal decomposition of UF-HC, is graphically presented in Figure 7. It is clear that temperature *T_onset_* has an increase trend in a linear fashion, as the heating rate increases. Other characteristic temperatures (*T_p_*, *T*_5_, *T*_10_, *T*_30_, and *T*_50_) are also subject to the same law, regarding the influence of the heating rate on their behavior (Figure S2, Supplementary Material). The obtained intercept values (Figure 7 and Figure S2) correspond to the limiting case (through extrapolation procedure), when *β* → 0 K/min. So, the dependence of these specific temperatures on the heating rate (*β*) is quantitatively expressed through established series of linear equations (Figure 7 and Figure S2), where diversity in linear regression coefficients can be observed. Since the value of *T_sh_* also shifts towards higher temperatures along with *T_p_*, it is obvious that a strong influence exists of the heating rate on the thermal decomposition process of UF-HC, where the parallel reactions may take place. The predominance of one of these reactions can be determined by the value of the selected heating rate.

As for the parameter *CPI* (the comprehensive performance index), directly abstracted from TG and DTG results (which primarily refers to the stage III; Figure 5), it shows an increase in the value, with an increase in heating rates (Table 2), where *CPI* is significantly improved with an increasing heating rate. Consequently, a better thermo-chemical performance of UF-HC is achieved at higher heating rates (larger *CPI* means a better suitability of the process).

**Figure 8:**
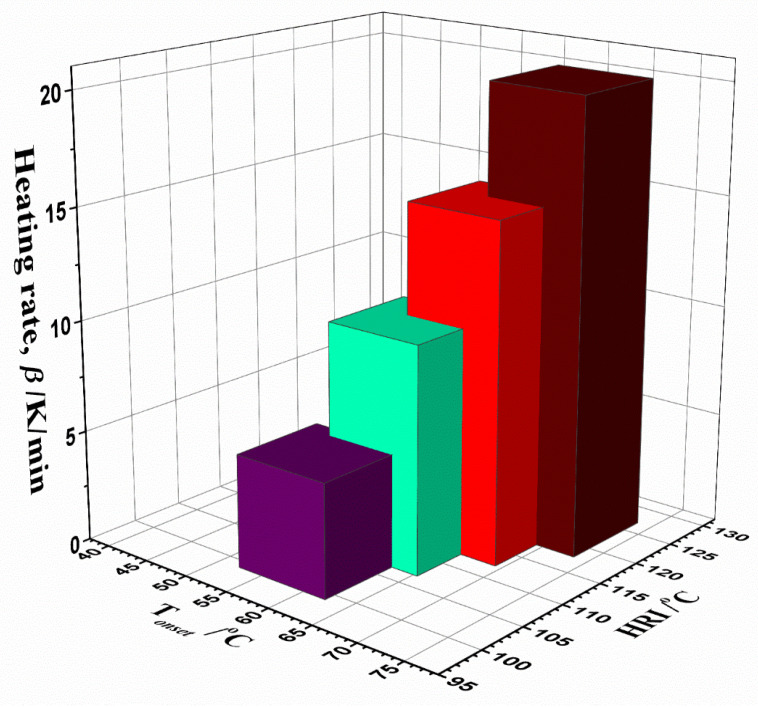
3D *T_onset_*–*HRI*–*β* body feature dependency, for the non-isothermal decomposition process of UF-HF composite.

Namely, the rise in both values, *R_max_* and *CPI*, at high heating rates (Table 2), improves the decomposition performance of UF-HC. Considering *HRI*, the presented *HRI* values are almost twice as high as those for *T_onset_*, and the largest jump in the *HRI* value is observed, when moving from 5.1 K/min to 10.2 K/min (for Δ(*HRI*) = 7.88 °C), while the ultimate change amounts to Δ*_u_*(*HRI*) = 18.88 °C (from 5.1 K/min to 20.2 K/min) (Table 2). So, there is a large impact of heating rate on the thermal resistance of UF-HC. The composite material already shows a higher thermal resistance at lower heating rates, and this resistance increases more and more as the heating rate increases (enhancement of *HRI*) (Table 2). These results are consistent with the temperature hysteresis observed in the thermo-analytical results. The influence of heating rate on both parameters, observing simultaneous changes in the *T_onset_*–*HRI* plane, can be seen in the 3D *T_onset_*–*HRI*–*β* body feature dependency shown in Figure 8. The obtained results show that the thermal characteristics of the UF-HC composite are incomparably better than in the case of pure UF conventional resin (polymer matrix) [98]. In addition, as the heating rate increases, the *IPDT* increases more and more, reaching the highest value at *β* = 20.2 K/min (Table 2).

The values of integral procedure decomposition temperature (*IPDT*) regarding the heating rate are located within the high-temperature regions. Taking these facts into account, it was shown that the synthesized composite exhibits a greater thermal stability (under similar experimental conditions) than the epoxy resin [40], urea–formaldehyde cellulose (UFC) composite [92], and pristine polyurethane (PU) retardant, as well as the polyurethane (PU)/hyper-branched nitrogen–phosphorous–silicon (PU/HBNPSi) hybrid composites [99].

### 3.6. Isoconversional Kinetic Analysis and Mechanistic Predictions of UF-HC Thermal Decomposition

**Figure 9:**
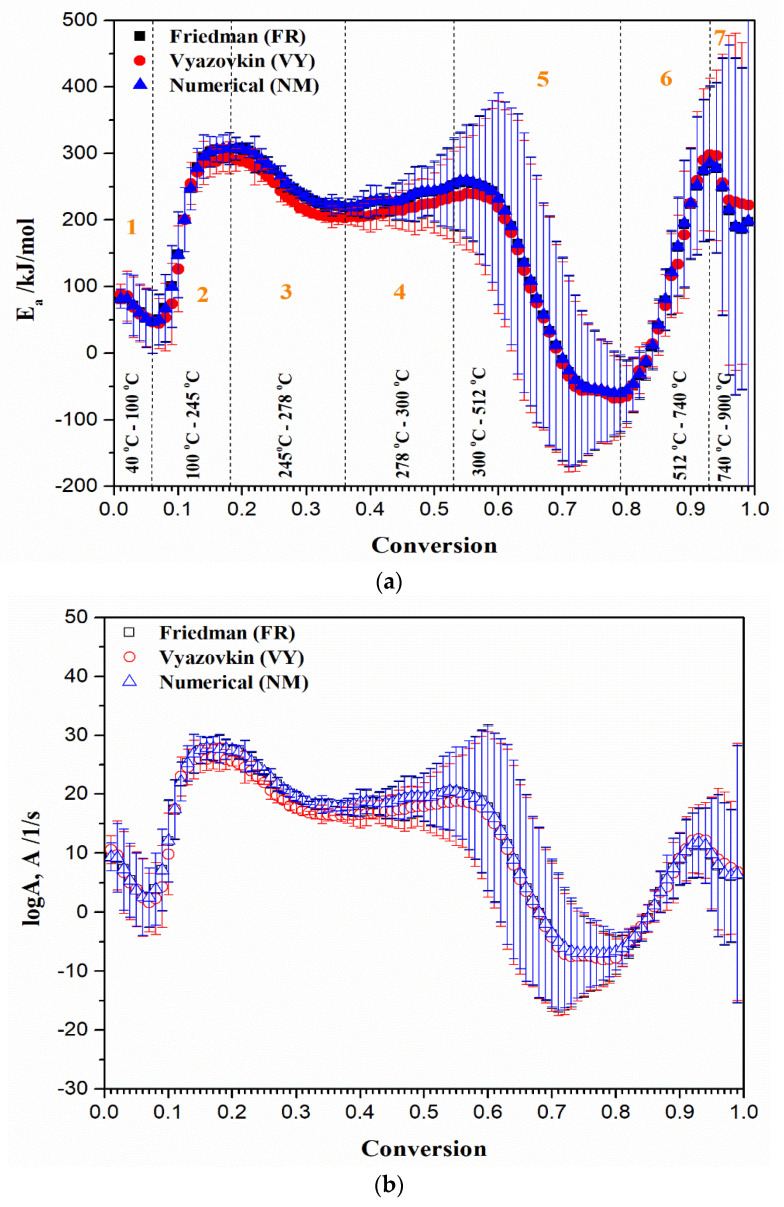
Conversion-dependent kinetic parameters estimated for non-isothermal thermal decomposition of UH-HC composite, by different isoconversional (model-free) methods: (**a**) *E_a_* = *E_a_*(conversion), and (**b**) *logA* = *logA*(conversion) plots, respectively. Corresponding values of standard deviations for the obtained kinetic parameters are presented in the form of vertical error bars.

Figure 9a,b show the conversion-dependent effective activation energy (*E_a_*) and logarithm of the pre-exponential factor (*logA*), for the non-isothermal thermal decomposition process of the UF-HC composite. From the obtained results, it can be seen that both kinetic parameters exhibit significant variation with conversion (α), indicating a strong kinetic complexity of the process. It can be observed from Figure 9a that seven different regions of reactivity exist, and they occupy different temperature zones, marked from “1” to “7”. These reaction zones are characterized by specific variations of both kinetic parameters, with progress of the process. It is worth noting that all the applied isoconversional (model-free) methods show an identical variation of effective activation energies and pre-exponential factors with conversion, and give very similar values of kinetic parameters. Likewise, in the area of higher conversions, for temperatures above 370 °C, the appearance of negative values of kinetic parameters was observed, especially for *E_a_*, which even reaches the value of −67.8 kJ/mol, observing the VY method. This phenomenon will be considered later, during the discussion of the presented results. However, in order to gain a deeper insight into the mechanistic profile of a given process, based on the data obtained from *E_a_* = *E_a_*(conversion) and *logA* = *logA*(conversion) plots, it is necessary to interpret each reaction region (Figure 9a), which would have physical meaning, and real consequences, arising from previously obtained results. Therefore, the general mechanistic picture (reactivity landscape) of the studied process would consist of the following items:

- For the first (1) region (for α = 0.01–0.06 and *T* = 40–100 °C) (Figure 9a) considering estimated values from the numerical optimization (NM) method): There is decrease in the *E_a_* value, from 81.61 kJ/mol to 46.56 kJ/mol. This reaction zone corresponds predominantly to the initial mass loss of the sample, and can be attributed to the dehydration of resin and/or some weaker interactions present in the current macromolecular structure [57]. The mean effective activation energy value for the actual zone (*E_a_*_(*mean*1)_) amounts 65.56 kJ/mol. This is a reasonable value, since dehydration energy covers values between 60 kJ/mol and 100 kJ/mol;
- For the second (2) region (for α = 0.07–0.18 and *T* = 100 °C–245 °C) (Figure 9a): The reaction zone is characterized by a jump in the value of effective activation energy, from 49.77 kJ/mol to 307.48 kJ/mol. In this part of the decomposition process, the mean effective activation energy value (*E_a_*_(*mean*2)_) of 217.28 kJ/mol was obtained. This event can be attributed to the conversion of methyl ether functional groups into methylene functional groups, with the release of free formaldehyde. The *E_a_* value estimated for the current conversion path may be also dependent on the F/U molar ratio, where it was reported that *E_a_* increases with an increasing F/U molar ratio (for F/U molar ratio of 1.25, the *E_a_* value of 287.2 kJ/mol was reported) [100]. Therefore, the obtained values of effective activation energy for the observed conversion path are quite realistic, considering the F/U molar ratio used here;
- For the third (3) and fourth (4) regions (for α = 0.19–0.36 and *T* = 245–278 °C, and for α = 0.37–0.53 and *T* = 278–300 °C, respectively) (Figure 9a): The two “joined” regions are characterized firstly by decreasing the value of *E_a_* from 307.78 kJ/mol (α~0.19) to 218.94 kJ/mol (α~0.36/0.37), and then by the gradual increase in the value of *E_a_*, from 220.60 kJ/mol (α~0.37/0.38) to 258.55 kJ/mol (α~0.53/0.54). These regions may include parallel temperature overlapping competitive reactions, in the multi-step complex reaction mechanism of UF-HC thermal decomposition. Namely, considering these reaction zones, the balance between bond breaking and cross-linking events coexists with each other. Within the third (3) region, the cleavage of C–N linkages to produce volatiles containing nitrogen should be expected [101], so that, probably, there are losses of chemical entities related to the reduction in N, O, and H; Further, a gradual increase in the *E_a_* value in the fourth (4) region may include the decomposition of residual lignin (arises from HC bio-filler), which could produce H_2_, that acts as an oxidizer and hydrogen donor for resin further decomposition. The current reaction zone involves the decomposition of UF resin-releasing products (mostly volatile products emitted during the non-isothermal decomposition of the resin). Considering the actual process reactivity zones, the UF-HC sample has lost the highest percent of its initial mass, including the largest share of volatiles in its further decomposition. The mean effective activation energy value for these zones (*E_a_*_(*mean*3+4)_) amounts to 246.92 kJ/mol. This value is in good agreement with the range of *E_a_* values, obtained for UF resin decomposition (150 kJ/mol–300 kJ/mol) [102];
- For the fifth (5) region (for α = 0.54–0.79 and *T* = 300–512 °C) (Figure 9a): This part of the process is characterized by a drastic drop in the effective activation energy (*E_a_*), which goes from approx. ~256.60 kJ/mol up to the lower values of *E_a_* until ~–60.53 kJ/mol. This *E_a_* area represents the transition from stage III to stage IV (the HC instability region) (Figure 5), in which decomposition of cellulose residues from HC mostly occurs, and also to the continued decomposition of biomass lignin. Namely, this part of the UF-HC thermo-chemical conversion is characterized by a primary charring process, where a fair amount of aromatic compounds is produced. Considering the thermo-analytical profiles of UF-HC decomposition shown in Figure 5, the main reinforcements arise from, firstly, stage IV, and then, and finally, stage V, where they slowly pace. Namely, the contribution of UF is minor, because, as can be seen from the TG curve of the UF-HC sample (Figure 5), the UF resin has lost the majority of its mass at an earlier stage, and here, only the release of volatiles from its further decomposition takes place, in a very slow rate manner. Therefore, chemical reactions which probably occur between UF resin and biomass residual components (primary cellulose-Cell) during the heating of the composite in earlier stages create an a more stable material thermally, at the end of this reaction region (at α ~ 0.79, Figure 9a). In other words, it can be said that the UF resin increases the thermal stability of lignocelluloses components left behind in HC, as it renders a decrease in their mass loss rate at the higher temperatures. However, the appearance of some negative *E_a_* values was observed, as we approach at the end of this part of the process. This phenomenon has both a mathematical and a physical explanation, because the negative value means that by increasing the temperature of the process, the rate constant then decreases (the negative dependence on the temperature), and this behavior represents a non-Arrhenius addiction (it is possible that a reversible reaction exists, which should be justified by two reaction paths, each of which has a positive activation energy, *E*). Namely, it is possible that a reaction mechanism exists, which is a ‘composite’ of the several elementary reaction steps, which have a negative activation energy. It can be assumed that a rapid pre-equilibrium occurs, which is exothermic in the first reaction step, followed by a second reaction step, which has a low positive activation energy value. In this mechanism, the net temperature dependence of the rate will be negative, i.e., the rate decreases with a decreasing temperature, and that is because the equilibrium constant (*K_eq_*) for the first reaction step decreases with an increasing temperature. This can be expected, considering the existence of exothermicity, related to stage IV, shown in Figure 5. However, this also should be linked with thermodynamic feasibility, through the possible occurrence of intermediate species. Namely, the overall decrease in the rate of the process may implicate that a negative *E_a_* exists in the current case, when reduction is caused by the decrease in the intermediate specie, as the process temperature is increased;
- For the sixth (6) region (for α = 0.80–0.93 and *T* = 512–740 °C) (Figure 9a): The region is characterized by rapid increase in the *E_a_* value, from approx. ~–56.29 kJ/mol up to ~286.23 kJ/mol (at α ~ 0.93). The current reactivity region is strictly connected with the pyrolysis of the carbon framework. The removal of oxygen functional groups probably occurred previously, within the (5) region. The “oxidized” graphite (Figure 3) has a high level of oxygen groups, which requires a lower level of energy for its decomposition, compared to the graphene and graphite. So, higher *E_a_* values are transferred to a high temperature zone (Figure 9a), since graphene demands a greater amount of thermal energy for the breaking of sp^2^ hybridized carbon atoms, ordered by covalent bonds in the hexagonal carbon framework. On the other hand, graphite, as the most thermodynamically stable carbon material, demands even more thermal energy, due to its strong 3D carbon network, consisting of a large number of graphene stacked layers, held by additional van der Waals forces [103];
- For the seventh (7) region (for α = 0.94–0.98/0.99 and *T* = 740–900 °C) (Figure 9a): Finally, the last region is characterized by decrease in the *E_a_* value, from approx. ~279.07 kJ/mol, up to ~187.06 kJ/mol. This reactivity region of UF-HC can be attributed to the thermal conversion process, where the majority of carbon atoms are converted from graphitic sp^2^ to non-graphitic sp^3^ carbon material. In this ultimate stage, there is a high probability that most of the oxygen functional groups from graphene oxide (GO) have been removed, during the reduction from GO.

#### Emergence of Kinetic Compensation Effect (KCE) During UF-HC Decomposition Process and Its Analysis

**Figure 10:**
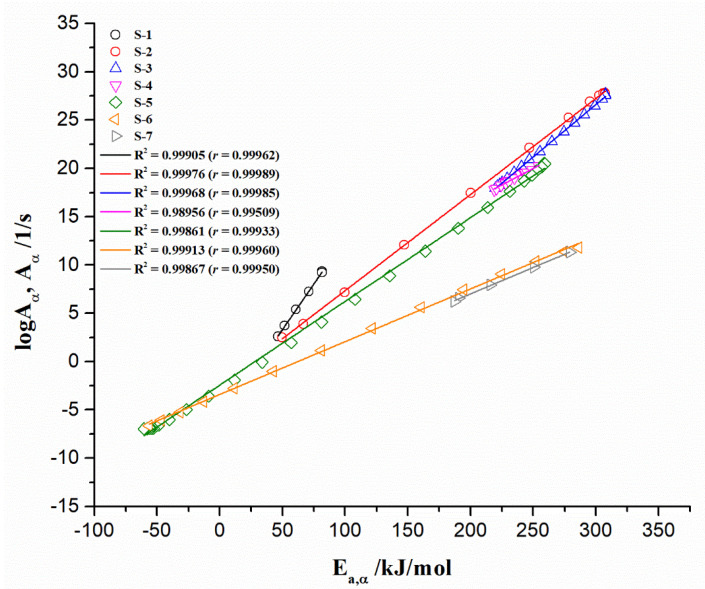
The KCE “branches” plots (S-1–S-7) derived for non-isothermal decomposition process of UF-HC composite.

The data *logA*_α_ and *E_a_*_,α_ were plotted to gain further insights into UF-HC thermal decomposition reactions, under different pyrolysis temperature regions. Figure 10 shows the linear relationships between kinetic parameters (*logA*_α_ and *E_a_*_,α_), estimated from the numerical optimization method (NM) (similar plots were obtained by other two isoconversional methods), and associated with stages “1–7” in Figure 9 (designated as S-1–S-7). This phenomenon is attributed to the kinetic compensation effect (KCE), where S-1–S-7 represents the KCE “branches” (Figure 10).

All the plots exhibit high values of Adjusted R-squared (R^2^) (also the values of Pearson’s correlation coefficient (*r*)) improving the existence of KCE, from the isoconversional (model-free) method/model. Table 3 lists the values of KCE coefficients (*a* and *b*), iso-kinetic rate constants, and iso-kinetic temperatures, related to each individual stage (S-1–S-7), and considering different conversion areas (Δα) (Figure 9).

From the results presented in Table 3, a strong KCE was obtained for the first three “branches” (S-1, S-2, and S-3) (high *r* values, above *r* = 0.99960), while for others, a somewhat weaker KCE was noted. Likewise, it can be seen that for branches S-1, S-2, and S-3, the calculated iso-kinetic temperatures (*T_iso_*) are within the experimental temperature range (Table 3), indicating that the results arising from the used isoconversional kinetic methods/models are the most suitable for the first three decomposition stages (S-1–S-3). This is also related to the magnitude size of standard deviations (errors), obtained during the calculation procedure of kinetic parameters by isoconversional methods. It can be seen that these errors are small and they are within the permissible limits for stages S-1, S-2, and S-3 (Figure 9a,b), while for stages S-4, S-5, S-6, and S-7, the emergence of high standard deviation values take place. However, it should be noted that the reliability of KCE is dependent on parameters determined from kinetic equations, consistent with the classical Arrhenius equation. Thus, any deviation from Arrhenius behavior introduces additional unreliability in the values of kinetic parameters, obtained by the isoconversional (model-free) approach. Consequently, the possibility of confronting isoconversional and compensation temperature (iso-equilibrium temperature) relationships arises (see later).

**Figure 11:**
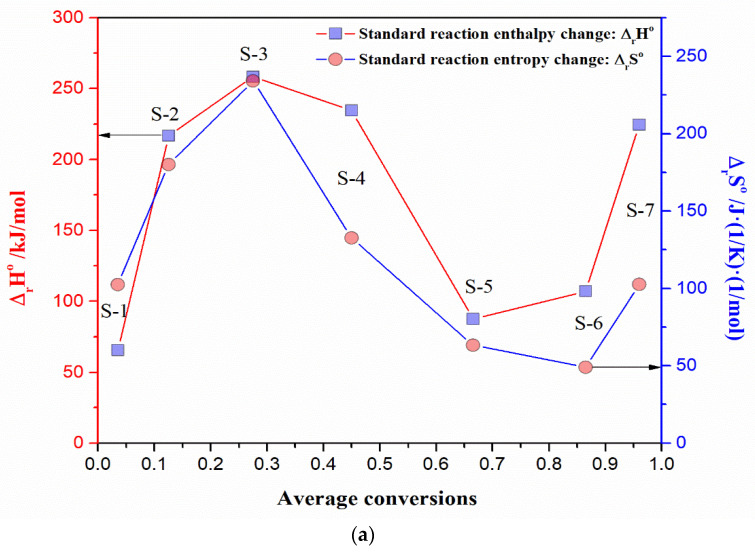

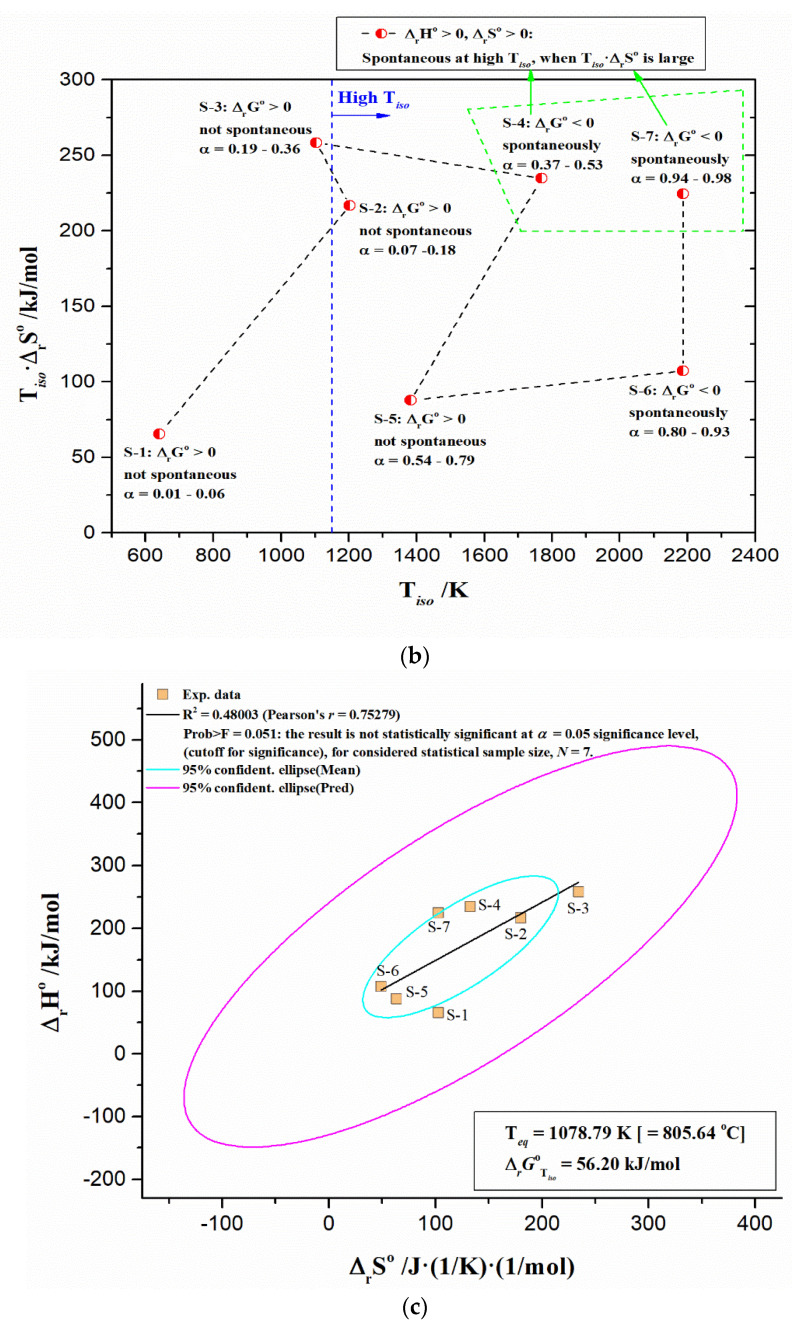
(**a**) Dependency of Δ*_r_H*° and Δ*_r_S*° on average conversions (α*_avg_*) regarding S1–S-7 KCE “branches” (Figure 10), (**b**) product dependency *T_iso_*·Δ*_r_*S° on the iso-kinetic temperature (*T_iso_* in K), regarding every considered stage (S-1–S-7), and in connection with the change in the standard Gibbs free energy of reaction (Δ*_r_G*°) (Δ*_r_G*° = Δ*_r_H*° − *T_iso_*·Δ*_r_*S°), and (**c**) Δ*_r_H*° − Δ*_r_S*° plot, related to thermodynamic data (Equation (13)), obtained for S-1–S-7 KCE “branches” (Figure 10) (the statistical sample size is *N* = 7).

The true KCE for stages S-1–S-3 probably exists, since this was proven through the use of more rigorous statistical analysis with the application of 95% confidential ellipses (mean and predicted) through enveloping experimental data, for all the reaction stages (S-1–S-7). These results are presented in Figure S3a–g (see Supplementary Material). It can be observed, that only for S-1–S-3, the infinitely elongated ellipses were obtained, whereby all the experimental points are located within both types of confidential ellipses. However, the confidential range depends on the size of statistical sample (*N*) (number of experimental points), so, for the experimental data points obtained for stages S-1–S-3, the number of observations causes the mean to become better and better estimated, leading to smaller and smaller confidential ellipses (contains smaller and smaller proportion of actual data (Figure S3a–g). Based on Equation (13), corresponding pairs of thermodynamic parameters (Δ*_r_H*° and Δ*_r_S*°) were calculated, for each of the KCE “branches” in Figure 10. Figure 11a shows dependencies of the change in the standard reaction enthalpies (Δ*_r_H*°) and the change in the standard reaction entropies (Δ*_r_S*°) on the average conversions (for each of the stages, from S-1 to S-7, the average values of the conversion in a given Δα range (Table 3) were taken into account).

From Figure 11a, there is a complex variation in both Δ*_r_H*° and Δ*_r_S*° values with α*_avg_*, considering the position of every KCE “branch”. However, both Δ*_r_H*° and Δ*_r_S*° are positive during the entire process with a continuous change in *T_iso_*, which means that energy is needed from outside of the system to reach its transition state, and also pushing towards randomness or disorder (positive Δ*_r_S*° values indicate that decomposition in the activated state has a less organized structure than before thermal disruption, but do not signify alone that the reaction is spontaneous). The latter indicates that as the process progresses, the entropy increases as the state of the virgin material changes from a solid to a liquid, and then to a gas phase. This especially applies to the S-2, S-3, and S-4 stages, with high positive values of Δ*_r_S*° (Figure 11 a)), suggesting the UF resin transition in UF-HC, from rigid to a semi-rubbery state, and then the breakdown of the polymer structure of the resin, with the release of gaseous products. Considering the spontaneity of the process by different stages, Figure 11b represents the dependence of *T_iso_*·Δ*_r_*S° on iso-kinetic temperature (*T_iso_*), regarding S-1–S-7. It can be observed that *T_iso_* has a strong influence on the Δ*_r_G*° value. So, during UF-HC thermal decomposition, the change in Δ*_r_G*° depends upon the change in Δ*_r_H*° and Δ*_r_S*°, according to Δ*_r_G*° = Δ*_r_H*° − *T_iso_*·Δ*_r_*S° (Figure 11b). Based on the obtained results, it can be seen that only in the case when both Δ*_r_H*° and Δ*_r_S*° are positive, Δ*_r_G*° will only be negative above a certain “threshold” temperature, so it can be said that the process stage is only spontaneous at a high *T_iso_* value. This specifically applies to stages S-4 and S-7 (when the term *T_iso_*·Δ*_r_*S° is large) (Figure 11b). This means that the additional disruption of residual lignocellulosic components in HC with the formation of liquid (volatile) products, as well as the release of gaseous products within S-4, requires a noticeable increase in temperature of the system, conditioning the shift of *T_iso_* to higher values. The same goes for the S-7, which is connected with the thermal conversion of graphitic sp^2^ into non-graphitic sp^3^ carbon material (see discussion above). The latter requires the input of a large amount of energy, moving *T_iso_* towards extreme values. Regarding the stage S-5, where exothermicity occurs, there is Δ*_r_G*° > 0 (not spontaneous) (Figure 11b), where spontaneity can proceed in a given condition, only for the reaction existing in a complex mechanism that takes place backwards, or under other circumstances discussed earlier (namely, the heat and mass transfer combined effects may be involved here) (see also the discussion above).

From established thermodynamic parameters (Δ*_r_H*° and Δ*_r_S*°), the enthalpy–entropy compensation plot between corresponding pairs of Δ*_r_H*° and Δ*_r_S*° values (Δ*_r_H*° − Δ*_r_S*° plot) was constructed. This plot is shown in Figure 11c. Based on the statistical analysis, a poor linear correlation was found between the thermodynamic parameters, taking into account an |*α*| = 0.05 significance level. Therefore, the derived result is not statistically significant at |*α*| = 0.05, for the considered statistical sample size of *N* = 7 (Prob > F = 0.051). So, the obtained value of the iso-equilibrium temperature (*T_eq_* = 1078.79 K) (Figure 11c), is actually “fake”, indicating that thermodynamic (enthalpy–entropy) compensation does not exist (so, for *T* > *T_eq_*, the activation loses its meaning). However, it should be noted that only for stage S-1 (referred to dehydration of UF resin), the value of *T_iso_* is lower than the value of *T_eq_* (Table 3), while in all the other cases, it was valid *T_eq_* > *T_iso_*. So, for the studied thermal decomposition process, the kinetic compensation effect (KCE) exists, but extra-thermodynamic information which would be obtained from the isoconversional kinetic approach is completely lost (*true* enthalpy–entropy compensation does not occur). Therefore, the real KCE is primarily driven by the resin dehydration pathway [104], throughout thermally induced conversion. Summarizing these results so far, KCE represents “isentropic” equilibration (it entails no heat and/or mass transfers, and no change in Δ*_r_S*°) of kinetic parameters at isoconversional (α = *const*.) temperature, *T_iso_* (Table 3), close to the *T_eq_*.

### 3.7. Model-Based Kinetic Results—Decrypting Entire Reactions Mechanism for UF-HC Decomposition Process

The model-based kinetic approach is usually based on the selection of the reaction model a priori. The first assumption for the model-based approach states that the process consists of several elementary reaction steps, and the reaction rate of each step can be described by its own kinetic equation, which can be presented in the following form:

(17) $${\left(\frac{d\alpha }{dt}\right)}_{j}={\left[\frac{d\left(a_{j}\to \;b_{j}\right)}{dt}\right]}_{j}=A_{j}\cdot exp\left(-\frac{E_{j}}{RT}\right)\cdot f\left(a_{j},b_{j}\right),$$

depending on the concentration of the initial reactant *a_j_*, the concentration of product *b_j_*, the pre-exponential factor *A_j_*, and the activation energy *E_j_*, specific only for the reaction step, with number *j*. In this study, kinetic computations were performed using the Kinetics Neo software (Version 2.7.0.11; Build date: 21 January 2024).

Within this software, reaction models are expressed as *f_j_*(*a_j_*,*b_j_*), where *a_j_* and *b_j_* stand for normalized concentrations of reactant and product, respectively, where the latter is usually presented in the form of (1 − *a_j_*) (or expressed via conversion quantity by various forms of function *f*(α), Table 1). During the calculation of multi-step kinetic models, each elementary step species has its own reactants extents, *a*, *b*, *c*, *d*, *e*, ….

The second assumption for the model-based approach expresses that all kinetic parameters, such as the activation energy, pre-exponential factor, reaction orders, and reaction models, are assumed constant during the reaction progress, for every individual reaction step. The third assumption for the model-based kinetic approach takes into account the total thermo-analytical signal, expressed through Equation (18) as follows:

(18) $$m=m_{o}-∆m\cdot \left[\sum_{j=1}^{n}c_{j}\int {\left(\frac{d\left(a_{j}\;\to \;b_{j}\right)}{dt}\right)}_{j}dt\right],$$

is the sum of signals of single reaction steps (where *m_o_* is the initial mass, ∆*m* represents the total mass change, “*n*” represents the total number of reaction steps, and *c_j_* is the contribution of the *j*-th reaction step); the effect of each step is calculated as the reaction rate multiplied by the effect of this step (for example, enthalpy change or the mass loss). In the case where a single-step process exists, both methods (model-free and model-based) give the results with the same kinetic parameters, which are fixed for the model-based method, or almost constant for the isoconversional method, during the considered process. However, for a complex (multi-step) process, where the kinetic mechanism is changing, a large difference may appear in the interpretation of results related to these two methods. For isoconversional methods, a change in kinetic model is described by the continuous change in kinetic parameters with an increase in α. But in the case of the model-based kinetic approach, the change in kinetic mechanism is simulated by the several reaction steps, with its own kinetic triplets.

The indicated kinetic complexity of the studied thermal decomposition process is established by the application of the various isoconversional methods (Figure 9), where it was clearly seen that current process consists of several reaction steps. However, it was not possible to determine how they were distributed, whether in parallel and independent steps, whether there is a consecutive mechanism, and similar cases. The kinetic complexity of the observed process can be seen from the constructed Master plot (*f*(α)/*f*(0.5) curves [105,106], estimated from the numerical (NM) and Vyazovkin’s (VY) model-free (isoconversional) data (see Figure S4a,b, Supplementary Material), which exhibit identical complex shapes, with the appearance of several peaks at different conversions (α). These plots confirm the multi-step nature of the UF-HC decomposition process, which requires the elucidation of the kinetic mechanism of the entire process, using another approach, which is the direct application of the model-based method. The decomposition process of polymer composites strongly depends on decomposition reactions that occur in the condensed phase and kinetic analysis permits to model the overall decomposition of a material, taking into account multi-step processes (if any). Note that those steps can be independent, parallel, competitive, or consecutive. This approach provides the decomposition pathway of the material studied. The importance of this analysis lies in the fact that it can provide sufficiently comprehensive features to achieve a good assessment on the mechanism of decomposition of a complex system, such as UF-HC, which can be used as a thermally stable adhesive. Thermal decomposition may vary with different forms of UF-based adhesives, and differs from one system to another, making generalization is difficult. The thermal decomposition of UF resin reinforced by fillers depends on the chemical nature of the filler, as well as the resin used in the process of synthesis. Therefore, the aforementioned kinetic approach provides useful information on the decomposition pathway of the studied material, regarding how and at which temperature the chemical reactions, such as the thermo-conversion of resin, occur. Finally, the output of this kinetic analysis provides the proposed most-relevant decomposition mechanism/reaction scheme. Furthermore, the established methodology for the characterization of the thermal decomposition of investigated material permits one to determine the main kinetic parameter of decomposition, namely “the activation energy of individual reaction step, *E*”. This corresponds to the minimum energy necessary to start an individual chemical reaction. Depending on the number of decomposition steps and on the current study, the multiple-step kinetic model can be used, and it can be more or less precise, which depends largely on the quality of the (‘best’) fit between experimental and numerical mass loss curves (TG-signals).

Over multiple rounds of calculation cycles, and optimization checking points for model accuracy, the aftermath of optimized kinetic models derived for each reaction step exists, so, the reaction scheme with code p:, Model was proposed. This scheme includes the existence of three independent single-step reactions and one consecutive reaction step, presented through Equations (19)–(22) as follows:

(19) $$A\;\overset{Fn}{\to }\;B$$

(20) $$C\;\overset{An}{\to }\;D\;\overset{Cn}{\to }\;E$$

(21) $$F\;\overset{F2}{\to }\;G$$

(22) $$H\;\overset{Fn}{\to }\;I,$$

with kinetic models sequent line F*n*A*n*C*n*F2F*n* (Table 1). In Equations (19)–(22), *A*, *C*, *F,* and *H* represent the reactants, *D* is the intermediate specie, while *B*, *E*, *G*, and *I* represent the products. The single-step reactions *A* → *B* and *H* → *I* are described by the *n*-th order chemical reaction (*n* ≠ 1) (F*n*), while the other single-step reaction *F* → *G* is described by the second-order chemical reaction (F2); the first reaction in the consecutive step (*C* → *D*) is described by *n*-dimensional nucleation (Avrami–Erofeev equation) (A*n*), while the second reaction in the consecutive step (*D* → *E*) is described by *n*-th order with autocatalysis (C*n*) (Table 1). It should be clearly emphasized that the series of reactions set above does not strictly follow temperature intervals on thermo-analytical curves, from room temperature (RT) to the maximum operating temperature of *T* ~ 950 °C. Each of the elementary reaction steps which are listed above occur in a precisely defined (-different) temperature intervals.

Corresponding rate-law equations, which include kinetic triplets for each reaction step of the proposed p:, Model, are given by Equations (23)–(27) as follows:

(23) $$\frac{d\left(a\;\to b\right)}{dt}=A\cdot a^{n}\cdot exp\left(-\frac{E}{RT}\right)$$

(24) $$\frac{d\left(c\;\to d\right)}{dt}=A\cdot n\cdot c\cdot {\left[-ln\left(c\right)\right]}^{\frac{\left(n-1\right)}{n}}\cdot exp\left(-\frac{E}{RT}\right)$$

(25) $$\frac{d\left(d\;\to e\right)}{dt}=A\cdot d^{n}\cdot \left(1+AutocatPreExp.\cdot (e)\right)\cdot exp\left(-\frac{E}{RT}\right)$$

(26) $$\frac{d\left(f\;\to g\right)}{dt}=A\cdot f^{2}\cdot exp\left(-\frac{E}{RT}\right)$$

(27) $$\frac{d\left(h\;\to i\right)}{dt}=A\cdot h^{n}\cdot exp\left(-\frac{E}{RT}\right),$$

where *AutocatPreExp*. ≡ *k_cat_*, and represents a weight factor (autocatalysis factor), it can be said that it is a frequency factor for the catalytic reaction path. Further, *a*, *b*, *c*, *d*, *e*, *f*, *g*, *h,* and *i* represent corresponding concentrations of the given chemical species involved. The appropriate mass balance equation (including reaction models established above) is expressed as follows:

(28) $$Mass=Initial\;Mass-Total\;Mass\;Change\times \left[Ctb.\left(a\to b\right)\int \left[\frac{d\left(a\;\to b\right)}{dt}\right]dt+Ctb.\left(c\to d\right)\int \left[\frac{d\left(c\;\to d\right)}{dt}\right]dt+Ctb.\left(d\to e\right)\int \left[\frac{d\left(d\;\to e\right)}{dt}\right]dt+\;Ctb.\left(f\to g\right)\int \left[\frac{d\left(f\;\to g\right)}{dt}\right]dt+Ctb.\left(h\to i\right)\int \left[\frac{d\left(h\;\to i\right)}{dt}\right]dt\right],$$

where *Mass* ≡ *m*, *Initial Mass* ≡ *m_o_*, and *Total Mass Change* ≡ Δ*m* in Equation (18), while *Ctb*. represents the contribution. Complete kinetic information regarding the proposed reaction mechanism presented by p:, Model, for the non-isothermal thermal decomposition of UF-HC, is summarized in Table 4.

The complete set of kinetic data obtained through the model-based method (Table 4) must be correctly interpreted, in order to obtain an accurate and realistic process mechanism. Therefore, the physicochemical interpretation of p:, Model is given in the lines below.

(a) The reaction step *F* → *G* (that occurs in temperature interval Δ*T* = RT − 180 °C/190 °C) described by a second-order chemical reaction (F2) (Table 4), is attributed to UF resin dehydration, where water and free formaldehyde (CH_2_O) are eliminated from the system. An activation energy of 58.750 kJ/mol corresponds to water evaporation and formaldehyde in the resin. The magnitude of the received *E* value is the benchmark for the increment/decrement of the condensation reactivity of UF resin. The contribution of this reaction step to the overall process is the smallest (~7.1% (Table 4)).

(b) The next essential process sequence is the consecutive reactions step: *C* → *D* → *E*. Namely, this sequential step represents the reaction contribution of cellulose (Cell) from hydrochar (HC). The first step in the sequential stage, *C* → *D* (that occurs in temperature interval Δ*T* = 200–400 °C), described by the nucleation and growth model (A*n* − *n*-dimensional nucleation; the Avrami–Erofeev model (Table 4)) [107] represents an accelerated step with a dimension of *n* ~ 0.181, indicating the one-dimensional growth of nucleation (reactivity) sites. It should be noted that intra-particle transport, which influences the rate during decomposition globally, can be caused by the increased particle size, subsequently provoking an increase in the temperature gradient inside particles. So, the cellulose chains can be gradually interrupted and decomposed locally, when the thermal energy is sufficient. Consequently, the influence of bio-filler on the direction of heat transfer tends to be irregular and random, and when the heat is transferred to the interior of the UF-HC sample, the relative position of bio-filler inside the tested sample may affect the heat transfer, thereby determining the reaction site (nucleation site).

The reaction step C → D can be attributed to the direct reaction pathway of furan formation from the cellulose (Cell) [108]. Thus, the furan formation from cellulose (Cell) requires a ring opening step, where glucose monomer (6-membered ring) is converted to a five-membered structure. According to this, the ring opening implies homolytic cleavage (with high activation energy which goes to 371.9 kJ/mol [109]) (obtained *E* value estimated for observed reaction step amounts ~352.831 kJ/mol (Table 4)), and the closure of the ring, while still attached to cellulose chain [110]. Namely, for temperatures above T = 200 °C, glycosidic bonds are cleaved homolytically with the simultaneous opening of the pyran ring (1C–5C) [110]. The linear intermediate then forms the furan ring by the 5O–radical attack at 2C, which is subsequently cleaved from the cellulose chain at 4C. In a considered reaction step, the furan represents the intermediate specie (*D*), which becomes the reactant in the next step. The contribution of C → D step to the overall process amounts to 16.8% (Table 4).

**Scheme 1:**
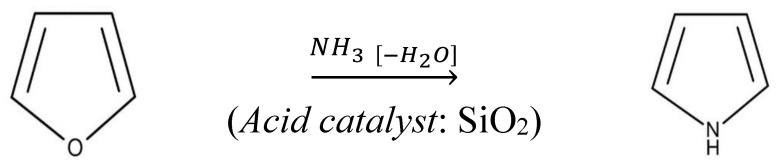
Production of pyrrole from furan and ammonia, in the presence of an acid catalyst (SiO_2_).

The second reaction in a consecutive mechanism (*D* → *E*) (that occurs in temperature interval Δ*T* = 300–500 °C) described by *n*-th order with autocatalysis (C*n*) hereby weighting factor *logk_cat_* = 1.45 (*k_cat_* = 0.282 × 10^2^) (Table 4) was attributed to the furan reaction with ammonia (NH_3_, generated by urea, already present inside the reaction system) in the presence of a solid acid catalyst as SiO_2_ to produce pyrrole (C_4_H_5_N; pyrrole(s) also includes its substituted derivatives—R) [111]. This reaction paves the way for the industrial production of pyrrole, and this is graphically represented in Scheme 1.

The kinetic model C*n* is the combination of *n*-th order and autocatalysis paths, which take place in a parallel manner. If it is assumed that the activation energies of these two paths are the same (=60.429 kJ/mol, Table 4), there is a difference in the pre-exponential factors of the two paths (values of *A* and *k_cat_* in Table 4). This is a simplified version of kinetic model C*nm* (namely, for *m* = 0, the reaction order (*n*) of *D* (furan) is *n* = 11.396 (Table 4), while pyrrole (*E*) plays a role as the first-order reaction). It should be noted that a high value of reaction order (*n* = 11.396) means (regarding to transition of reactant (in this case, it is *D*)) that the reaction rate will decrease faster. On the other hand, the accelerating magnitude is estimated based on the pre-exponential factors of the two paths. It can be observed that the pre-exponential factor for acceleration path (=0.282 × 10^2^) is higher than the pre-exponential factor for “deceleration” path (=0.127 × 10^2^) (it is twice the value) (Table 4), thereby increasing the yield of pyrroles. Namely, this reaction step represents a green protocol [112] for the production of N-heterocyclic compounds. In this stage, we have a huge consumption of ammonia, and its “trapping” in a described process segment. The contribution of *D* → *E* step to the overall process amounts to 19.8%. At the same time, observing the whole consecutive reactions step *C* → *D* → *E*, it represents the formation of nitrogenous heterocyclic compounds from the cellulose (Cell) (originating from HC). It should be emphasized that in the temperature range between 200 °C and 500 °C, the main gaseous decomposition products appear, such as HCN, NH_3_, CO, CO_2_, and H_2_O [113].

(c) Independent single-step reaction, marked as *A* → *B* (it occurs in temperature interval Δ*T* = 200–400 °C) is described by the *n*-th order reaction model (F*n*) (Table 4), and it can be attributed to the cleavage and breaking of methylene ether bridges, with an activation energy (*E*) of 164.933 kJ/mol (Table 4). The obtained activation energy for the current step corresponds to the formation of urea ions and methylolurea hemiformal (HF_n_) [114], which coincides with the disruption of the type I methylene ether bridge, in the presence of water vapor. The observed reaction step is associated with thermal dissociation into the urea compounds/derivatives [115,116]. The contribution of this reaction to the overall process amounts to 43.7%.

(d) Another independent single-step reaction, *H* → *I* (that occurs in temperature interval Δ*T* = 500–950 °C), described by the *n*-th order reaction model (F*n*) (Table 4), can be attached to the deoxygenation reaction of graphene oxide (GO) within the HC component in UF-HC, which is a chemically controlled reaction with an activation energy of 155.882 kJ/mol (Table 4) (thermal reduction) [117]. The obtained kinetics results are in good agreement with graphene-based filler with metals impurity case studies. This has applications in polymer composite materials and bio-based adhesives [118,119]. In this reaction, H_2_O, CO, and CO_2_ are liberated as gaseous products [120]. Also, this reaction step represents the carbon material modification pathway by a high-temperature-intensified process, making any existing graphite clusters smaller, and causing π-bonds to be broken, which may result in an increase in sp^3^ content. Considering the HC bio-filler component, sp^3^-bonded carbon is probably developing as cross-links in the final micro-structure at the end of the process, and most likely disturbs a well-ordered structure of carbon crystallites. The contribution of the observed reaction to the overall process amounts to 12.6 % (Table 4).

**Figure 12:**
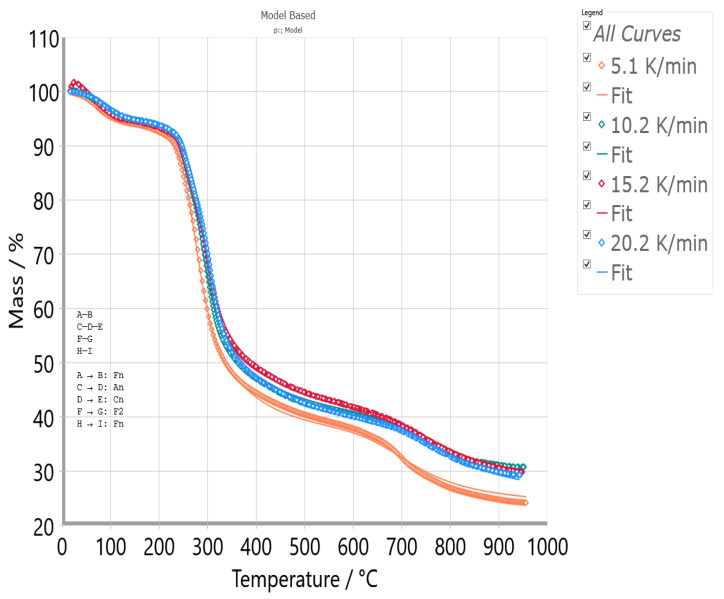
Comparison of model-based kinetic results (p:, Model) with the experimental TG-signals at the different heating rates (*β* = 5.1, 10.2, 15.2, and 20.2 K/min), for non-isothermal decomposition process of UF-HC (R^2^ = 0.99977).

Based on the proposed p:, Model scheme, the obtained kinetic results (Table 4) were used to compare them with the experimental TG-curves, at different heating rates. The results of fitting the model (p:, Model) with the experimental thermo-analytical data are shown in Figure 12.

From the obtained results of fitting, it is evident that the proposed model fits very well with the experimental TG-curves with high R^2^ values (=0.99977), and fully satisfies a quite decent prediction (namely, the R^2^ value should be around 0.99900 to give satisfactory predictions).

It should be noted that on the basis of the combination of kinetic models described above, which are in the framework of p:, Model scheme, there is an overlapping character of some reactions, in the considered temperature intervals (see above). Likewise, it is observed that reactions *A* → *B* and *C* → *D* are taking place simultaneously in the same temperature range, running in the parallel manners. So, steps that include reaction sequences like *A* → *B* and *C* → *D* → *E* appear to be in a competition, so that the favoring of some stage may be governed by the heating rate, in a determining rate-controlling step. The rate-controlling step estimates the slowest reaction step; so, it was found that at a high heating rate (~ 20.2 K/min), the rate constant for step *C* → *D* (*k_C_*_→*D*_ = 0.00352 1/s) is much higher than the rate constant for step *D* → *E* (*k_D_*_→*E*_ = 0.000643 1/s) (*k_C_*_→*D*_ >> *k_D_*_→*E*_), indicating that the step *D* → *E* (production of pyrroles) represents rate-limiting step. So, since the step *D* → *E* represents the rate-limiting step at a high heating rate, in this case, there will be a significant build-up of intermediate *D* (*C* is rapidly converted into *D*) (*furan*) (obtaining a high yield of furan), and then the slow building of *E* (the lower yield of *pyrrole*). On the contrary, at a low heating rate (~5.1 K/min), the values of rate constants are comparable (*k_C_*_→*D*_ = 0.000837 1/s > *k_D_*_→*E*_ = 0.000245 1/s), and the ratio of these rate constants amounts to *k_C_*_→*D*_/*k_D_*_→*E*_ = 3.42. In this case, the influence of heating rate is reflected by the properties of C*n* kinetic model. For the considered kinetic model, favoring one of two paths is conditional by the frequency factors (see Table 4), including *n*-th (furan) and first-order (pyrrole) types. Therefore, at the low heating rate (~5.1 K/min), the first-order generation of pyrroles dominates, but a high yield of pyrrole should not be expected, as in the previous case, for the furan production. Considering only two reaction steps, *A* → *B* and *C* → *D*, which take place in the same temperature interval (200–400 °C), at low heating rate (~5.1 K/min), the rate constant for step *A* → *B* (methylene ether bridge disruption and formation of methylolurea hemiformal (HF_n_)) is much higher than the rate constant for the step *C* → *D* (*k_A_*_→*B*_ = 0.00109 1/s >> *k_C_*_→*D*_ = 0.000837 1/s); so, in this case, the fractional yield of the product *B* depends on the ratio of the rate constants *k_C_*_→*D*_/*k_A_*_→*B*_, so it was obtained as *k_C_*_→*D*_/*k_A_*_→*B*_ = 0.77. It can be concluded that for the investigated reaction system, lower heating rates are recommended to achieve higher yields of urea compounds (see discussion above).

### 3.8. Statistical Fit Quality Comparison Between Used Methods/Models, for Kinetic Investigation Related to Decomposition Process of UF-HC

For the statistical analysis, besides the best coefficient of determination (R^2^) and the F-test (Fisher’s exact test), the sum of deviations squares (S^2^), mean residual (MR), and Student’s coefficient of 95% confidence intervals, are used in this work. Details about these statistical parameters can be found elsewhere [121]. Table 5 shows the comparative statistical analysis results of the different model-free (isoconversional) methods (Friedman (FR), Vyazovkin (VY), and the numerical optimization (NM) methods) and the model-based method, for the non-isothermal decomposition of UF-HC.

Considering the obtained values of statistical parameters (Table 5), there is the following order of methods/models, according to the quality of fitting the experimental data: p:, Model > Numerical (NM) > Friedman (VY) > Vyazovkin (VY). Among isoconversional methods, numerical optimization (NM) is superior in relation to FR and VY methods. So, the results estimated by the application of the last two isoconversional methods should be used with caution. However, looking at all the methods applied, the model-based method with proposed p:, Model scheme is the best reactions model (R^2^ > 0.99950 (for R^2^ = 0.99977) follows F = 1.000), for suitable explanations of all physicochemical phenomena, which occur during the non-isothermal decomposition process. The advantage of the model-based method incorporated into Kinetics Neo software (The NETZSCH Group Holding, Selb, Germany) (Version 2.7.0.11; Build date: 21 January 2024) represents the ability to implement successive optimizations with sub-sets of parameters prior to a final optimization with parameters that are close to the final values, and can construct complex reaction models, such as parallel, sequential, and competing reactions in the real time. The latter cannot be recognized by standard differential and integral isoconversional methods. Another important advantage of the model-based approach is the possibility that the reaction kinetic parameters can be fixed reliably by other considerations (not only by R^2^, but also with other much more reliable statistical criteria, such as the sum of deviations square and mean residual, with F-test priority (Table 5)); so, the probability of achieving a defendable model significantly improves. According to all the mentioned crucial indicators, model-free (isoconversional) models cannot achieve all these requirements so far. Therefore, the above-described framework for kinetics data extraction shows that the set of kinetic triplets listed in Table 4 are the most suitable for the rational decrypting of entire reactions, which appear during UF-HC decomposition.

In summary, the manufactured bio-composite based on UF resin (with low F/U) and biomass HTC-derived hydrochar (HC) (UF-HC) can be characterized by comprehensive properties as a green adhesive, which could be used commercially in the form of an eco-friendly adhesive. Therefore, based on the test results presented so far, they clearly show that the manufactured composite could be a promising material in the development of green adhesives within bio-composite fabrication, with improved physicochemical properties.

## 4. Conclusions

In this study, the synthesis of a bio-composite based on UF resin (with F/U = 0.80) and hydrochar produced from hydrothermal carbonization (HTC) of spent mushroom substrate (SMS) (used as the biomass feedstock) (marked with UF-HC) was performed. The physicochemical characterization of the obtained UF bio-composite was carried out using FTIR, XRD, and SEM techniques. The thermal stability analysis of the synthesized composite was performed using the data obtained from thermo-analytical measurements. The kinetic analysis of the thermal decomposition process of UF-HC was carried out using the data from non-isothermal thermal analysis measurements, in an inert (Ar) atmosphere (simultaneous TGA-DTG-DTA measurements at four different heating rates, as *β* = 5.1, 10.2, 15.2 and 20.2 K/min). Within the kinetic analysis framework, two approaches were implemented, including the model-free (isoconversional) methods (Friedman (FR), Vyazovkin (VY), and numerical optimization (NM)) and the model-based method (using the multivariate non-linear regression computational procedure). Based on the results and discussion, the following concluding points are drawn:

- Morphological characterization of the UF-HC by SEM showed enhanced hardness, in-creased the surface roughness, and most possibly enhanced the impact resistance. However, the adhesion strength depends on the applicable F/U ratio;
- Further, the structural analysis by FTIR and XRD techniques showed the presence of crystalline regions of UF resin with an aggregated crystalline region of cellulose type I, which was left behind from the biomass part, after the implementation of hydrothermal carbonization under mild conditions. Also, the presence of impurity inside UF-HC, in the form of crystalline SiO_2_ (quartz), was also observed. Furthermore, it was confirmed that UF-HC contains lattices with an oxidized face—graphene oxide—GO, confirming the presence of oxygen-containing functional groups;
- TGA-DTA measurements pointed to a highly complicated decomposition reaction profile for UF-HC. It comprises a number of parallel and consecutive reactions, occurring at the long-range of process temperatures. The inherent thermal stability analysis showed a high thermal resistance of UF-HC composite when subjected to thermal stress, which was clearly indicated by the *IPDT* parameter (the integral procedure decomposition temperature), which raised the value as the heating rate increased, to above 530 °C (with an average value of 545.41 °C). It was established that there is a large impact of heating rate on the thermal resistance of the UF-HC composite;
- The existence of a kinetic compensation effect (KCE) was found, but also the absence of thermodynamic (enthalpy-entropy) compensation. KCE appears as an “isentropic” equilibration of the kinetic parameters at isoconversional temperature, *T_iso_*, close to the iso-equilibrium temperature (*T_eq_* = 805.64 °C);
- The non-isothermal decomposition process of UF-HC having complex mechanistic scheme (p:, Model), consisting of three independent single-step reactions and one consecutive reactions step. The three independent single-step reactions are attributed to the following: (a) UF resin dehydration described by F2 model (Δ*T* = RT − 190 °C; H_2_O + CH_2_O releases), (b) cleavage and breaking of methylene ether bridges, with formation of methylolurea hemiformal (HF_n_), described by F*n* model (Δ*T* = 200–400 °C), and (c) deoxygenation of graphene oxide (GO), which represents a chemically controlled reaction (F*n* model) (Δ*T* = 500–950 °C). The consecutive reactions step consists of the following transformations: first, the direct reaction pathway of furan formation from cellulose, that includes a ring opening step, where glucose monomer (6-membered ring) is converted to a five-membered structure (the formed furan represents an intermediate specie, which becomes the reactant in the next step) (A*n* model: *n*-dimensional nucleation, Δ*T* = 200–400 °C), and in the second, the furan reaction with NH_3_, in the presence of a solid acid catalyst (SiO_2_) to produce pyrrole (C_4_H_5_N), described by the *n*-th order with an autocatalysis (C*n*) (Δ*T* = 300–500 °C);
- It was found that the heating rate represents a regulatory factor in determining the rate-controlling step. It has been shown that for the consecutive decomposition stage, a high heating rate promotes the production of intermediates—furan (the high yield of furan can be estimated), and the slower generation of pyrrole (the lower pyrrole yield may be predicted). On the other hand, it was found that a low heating rate favors the production of pyrrole, but with a moderate yield. Likewise, in this study, it was shown that lower heating rates are recommended, if higher yields of urea compounds with oligomeric chains are to be obtained;
- The synthesized bio-composite meets the requirements for a green adhesive, as an acceptable eco-friendly adhesive (in terms of reduced formaldehyde emission and ammonia, via the scavenger reaction pathways).

## Figures and Tables

**Table 1:** Kinetic model functions (in differential form of analytical kinetic functions, *f*(*α*)) used in this work, for computational procedure in the model-based analysis [48].

Model	Symbol	*f*(α)
Phase boundary-controlled reaction (contracting disk, 1D)	R1/F0	(1 − *α*)^0^
Phase boundary-controlled reaction (contracting area, 2D)	R2	2·(1 − *α*)^1/2^
Phase boundary-controlled reaction (contracting volume, 3D)	R3	3·(1 − *α*)^2/3^
Random nucleation, unimolecular decay law, and first-order chemical reaction	F1	(1 − *α*)
Second-order chemical reaction	F2	(1 − *α*)^2^
*n*-th order chemical reaction (*n* ≠ 1)	F*n*	(1 − *α*)*^n^*
Two-dimensional growth of nuclei (Avrami equation)	A2	2·(1 − *α*)[−ln(1 − *α*)]^1/2^
Three-dimensional growth of nuclei (Avrami equation)	A3	3·(1 − *α*)[−ln(1 − *α*)]^2/3^
*n*-dimensional nucleation (Avrami–Erofeev equation)	A*n*	*n*·(1 − *α*)[−ln(1 − *α*)]^1−1/*n*^
One-dimensional diffusion, parabola law	D1	1/2*α*
Two-dimensional diffusion, Valensi equation	D2	1/[−ln(1 − *α*)]
Three-dimensional diffusion, Jander equation	D3	(3/2)(1 − *α*)^2/3^/[1 − (1 − *α*)^1/3^]
Three-dimensional diffusion, Ginstling–Brounstein	D4	(3/2)/[(1 − *α*)^−1/3^ − 1]
Prout–Tompkins equation	B1	(1 − *α*)·*α*
Expanded Prout–Tompkins equation	B*na*	(1 − *α*)*^n^·α^a^*
First order with autocatalysis	C1	(1 + *k_cat_*·*α*)(1 − *α*)
*n*-th order with autocatalysis	C*n*	(1 + *k_cat_*·*α*)(1 − *α*)*^n^*
*n*-th order and *m*-power with autocatalysis	C*nm*	(1 − *α*)*^n^*·*α^m^*
Expanded Šestak–Berggren (SB) equation	SB*nmq*	(1 − *α*)*^n^*·*α^m^*·[−ln(1 − *α*)]*^q^*
Kamal–Sourour equation	KS	(*k*_1_ + *k*_2_·*α^m^*)(1 − *α*)*^n^*
Nakamura crystallization	Nk (A*n* + H–L)	*f*(*α*)·*K*(*T*), *f*(*α*) = *n*·(1 − *α*)[−ln(1 − *α*)]^1−1/*n*^, where for analytical dependence of the rate constant *K*(*T*), Hoffman–Lauritzen (H–L) theory is used (non-Arrhenius)
Šestak–Berggren crystallization or Sbirrazzuoli crystallization	(SBC/SC) (SB + H–L)	*f*(*α*)·*K*(*T*), *f*(*α*) = (1 − *α*)*^n^*·*α^m^*·[−ln(1 − *α*)]*^q^*, where for analytical dependence of the rate constant *K*(*T*), Hoffman–Lauritzen (H–L) theory is used (non-Arrhenius)

**Table 2:** Thermal stability characteristic parameters for UF-HC composite, determined at different heating rates (*β* = 5.1, 10.2, 15.2, and 20.2 K/min).

*β* (K/min)	*T_onset_* ^a^ (°C)	*T_sh_* ^b^ (°C)	*T_p_* ^b^ (°C)	*T_i_* ^a^ (°C)	*T_f_* ^a^ (°C)	*T*_5_ ^a^ (°C)	*T*_10_ ^a^ (°C)	*T*_30_ ^a^ (°C)	*T*_50_ ^a^ (°C)	*R_max_* ^b^ (%/min)	*CPI* (%^2^·min^−2^·°C^−3^)	*HRI* (°C)	*IPDT* (°C)
5.1	57.40	244.04	279.49	233.85	879.44	106.19	235.07	281.28	335.51	3.420	3.400 × 10^−7^	103.51	534.54
10.2	60.30	255.16	293.44	236.64	885.55	129.40	248.48	292.60	355.06	8.377	1.551 × 10^−6^	111.39	540.54
15.2	64.20	262.87	299.20	240.18	899.25	134.53	262.28	307.94	377.83	11.346	2.939 × 10^−6^	116.90	548.84
20.2	68.34	269.65	303.91	246.98	910.47	141.78	275.31	321.78	389.30	16.616	5.040 × 10^−6^	122.39	557.71

**^a^** Determined from TG-curves, recorded at the different heating rates (Figure S1—Supplementary Material). **^b^** Determined from DTG-curves shown in Figure 6.

**Table 3:** KCE parameters (*a*, *b*, *k_iso_*, and *T_iso_*) obtained for non-isothermal decomposition process of UF-HC composite.

Stage	Δα	*a* (s^−1^)	*b* (mol·(kJ)^−1^)	*k_iso_* (s^−1^)	*T_iso_* (°C)	Experimental *T*-Range/Effect	R^2^	*r*
S-1	0.01–0.06	−6.103 ± 0.173	0.188 ± 0.003	7.889 × 10^−7^	366.63	Inside ^a^	0.99905	0.99962
S-2	0.07–0.18	−2.609 ± 0.111	0.1 ± /	0.003	929.64	Inside ^a^	0.99976	0.99989
S-3	0.19–0.36	−5.941 ± 0.123	0.109 ± /	1.145 × 10^−6^	830.33	Inside ^a^	0.99968	0.99985
S-4	0.37–0.53	3.029 ± 0.409	0.068 ± 0.002	1.069 × 10^3^	1495.66	Outside	0.98956	0.99509
S-5	0.54–0.79	−2.441 ± 0.099	0.087 ± /	0.004	1109.37	Outside	0.99861	0.99933
S-6	0.80–0.93	−3.407 ± 0.072	0.055 ± /	0.0004	1913.74	Outside	0.99913	0.99960
S-7	0.94–0.98	−3.927 ± 0.227	0.055 ± /	0.0001	1913.74	Outside	0.99867	0.99950

^a^ Figure S1 (Supplementary Material).

**Table 4:** Kinetic parameters and contribution of each reaction step in proposed p:, Model, for non-isothermal decomposition process of UF-HC.

Step ^a^: *A* → *B*, Reaction Type: F*n*	
Activation Energy, *E* (kJ/mol)	164.933
Log(PreExp.), *logA*, *A* (1/s)	13.495
Reaction Order, *n*	3.438
Contribution	0.437
Step **^a^**: *C* → *D*, Reaction Type: A*n*	
Activation Energy, *E* (kJ/mol)	352.831
Log(PreExp.), *logA* (1/s)	31.472
Dimension, n	0.181
Contribution	0.168
Step **^a^**: *D* → *E*, Reaction Type: C*n*	
Activation Energy, *E* (kJ/mol)	60.429
Log(PreExp.), *logA*, *A* (1/s)	1.105
Reaction Order, *n*	11.396
Log(AutocatPreExp.), *logk_cat_*	1.450
Contribution	0.198
Step **^a^**: *F* → *G*, Reaction Type: F2	
Activation Energy, *E* (kJ/mol)	58.750
Log(PreExp.), *logA* (1/s)	6.761
Contribution	0.071
Step **^a^**: *H* → *I*, Reaction Type: F*n*	
Activation Energy, *E* (kJ/mol)	155.882
Log(PreExp.), *logA*, *A* (1/s)	5.534
Reaction Order, *n*	2.608
Contribution	0.126

**^a^** Each elementary step represents a distinct chemical change, and together they describe the overall process of how reactants are converted into products.

**Table 5:** Comparative statistical analysis (a statistical fit quality) between isoconversional methods (Friedman (FR), Vyazovkin (VY), and numerical optimization (NM)) and model-based method (p:, Model), for non-isothermal decomposition of UF-HC.

Method	Fit To	R^2^	Sum of dev. Squares (*S*^2^)	Mean Residual (MR)	Students coef. 95%	F-Test
Friedman (FR)	TG ^a^	0.97506	67,429.646	3.329	1.961	114.435
Vyazovkin (VY)	TG ^a^	0.97450	68,922.355	3.433	1.961	116.968
Numerical (NM)	TG ^a^	0.99712	7877.474	1.096	1.961	13.369
p:, Model	TG ^a^	0.99977	646.650	0.446	1.961	1.000

^a^ TG—thermogravimetry signal.

## Data Availability

The original contributions presented in this study are included in the article. Further inquiries can be directed to the corresponding author.

## Supplementary Materials

The following supporting information can be downloaded at https://www.mdpi.com/article/10.3390/polym17101375/s1: Figure S1: Thermogravimetric curves of non-isothermal thermal decomposition process of UF-HC composite, at the different heating rates (*β* = 5.1, 10.2, 15.2, and 20.2 K/min); Figure S2: Temperature dependence *T_p_*, *T*_5_, *T*_10_, *T*_30_ and *T*_50_ on the heating rate (*β*), for non-isothermal thermal decomposition process of UF-HC composite (the values of linear regression coefficients, are also provided (R^2^ and Pearson’s (*r*))); Figure S3: *logA*_α_ vs. *E_a_*_,α_ plots (a–g) with introduced 95% confidential ellipses (mean and predicted) regarding to all observed reaction stages (1–7), identified from isoconversional kinetic method (NM), and established through seven KCE “branches” (see the main text); and Figure S4: Master plots (*f*(α)/*f*(0.5) (a ≡ α) obtained from (a) numerical optimization (NM) and (b) Vyazovkin’s (VY) model-free (isoconversional) data, for the non-isothermal thermal decomposition process of UF-HC composite.

## Abbreviations

The following abbreviations are used in this manuscript:

UF	Urea–formaldehyde
MF	Melamine–formaldehyde
BG	Benzo–guanine
U	Urea
F	Formaldehyde
M	Melamine
FE	Formaldehyde emission
PLA	Polylactic acid
HTC	Hydrothermal carbonization
HHV	Higher heating value
HC	Hydrochar
SMS	Spent mushroom substrate
UF-HC	Urea–formaldehyde hydrochar
HRI	The heat-resistance index
IPDT	The integral procedure decomposition temperature
CPI	The comprehensive performance index
TGA	Thermogravimetric analysis
DTA	Differential thermal analysis
FTIR	Fourier transform infrared
SEM	Scanning electron microscopy
XRD	X-Ray diffraction
SMS-HTC	Spent mushroom substrate-hydrothermal carbonization
RM	Room temperature
ATR	Attenuated total reflectance
CrI	Crystallinity index
TG	Thermogravimetry
DTG	Derivative thermogravimetry
Q	Quartz
GO	Graphene oxide
Cell	Cellulose
MVarNLRM	The multivariate non-linear regression method
KCE	The kinetic compensation effect
IKP	The isokinetic point
UFC	Urea–formaldehyde cellulose
PU	Polyurethane
PU/HBNPSi	Polyurethane/hyper-branched nitrogen–phosphorous–silicon
HF_n_	Methylolurea hemiformal
FR	Friedman
VY	Vyazovkin
NM	Numerical optimization
