# Supplementary material for: Synthesis and Characterization of Bio-Composite Based on Urea–Formaldehyde Resin and Hydrochar: Inherent Thermal Stability and Decomposition Kinetics
Source: Polymers (Basel). 2025 May 16;17(10):1375. doi: 10.3390/polym17101375 (PMC12115115; doi:10.3390/polym17101375)
Supplement: Supplementary file 1 [file polymers-17-01375-s001.zip › Supplementary Material.pdf]

●*Supplementary Material*

●*POLYMERS*

Title: "**Synthesis and Characterization of Bio-Composite Based On Urea-Formaldehyde Resin and Hydrochar: Inherent Thermal Stability and Decomposition Kinetics**"

Authors: Bojan Janković, Vladimir Dodevski* (*Corresponding author*), Marija Janković, Marija Milenković, Suzana Samaržija-Jovanović, Vojislav Jovanović, and Milena Marinović-Cincović

Corresponding author affiliation: University of Belgrade, "Vinča" Institute of Nuclear Sciences – National Institute of the Republic of Serbia, Mike Petrovića Alasa 12-14, P.O. Box 522, 11001 Belgrade, Serbia

● *Supplementary Material Content – Results Section (S-1):*

Figure S1. Thermogravimetric curves of non-isothermal thermal decomposition process of UF-HC composite, at the different heating rates (β = 5.1, 10.2, 15.2, and 20.2 K/min)…………S-2

Figure S2. Temperature dependence T_p, T_5, T_{10}, T_{30} and T_{50} on the heating rate (β), for non-isothermal thermal decomposition process of UF-HC composite (the values of linear regression coefficients, are also provided (R^2 and Pearson's (r)))…………S-3

Figure S3. $logA_\alpha$ *vs*. $E_{a,\alpha}$ plots (**a**) – **g**)) with introduced 95 % confidential ellipses (mean and predicted) regarding to all observed reaction stages (1 – 7), identified from isoconversional kinetic method (NM), and established through seven KCE "branches" (see the main text)…………S-4

Figure S4. Master plots ($f(\alpha)/f(0.5)$) (a ≡ α) obtained from **a)** numerical optimization (NM) and **b)** Vyazovkin's (VY) model-free (isoconversional) data, for the non-isothermal thermal decomposition process of UF-HC composite…………S-5

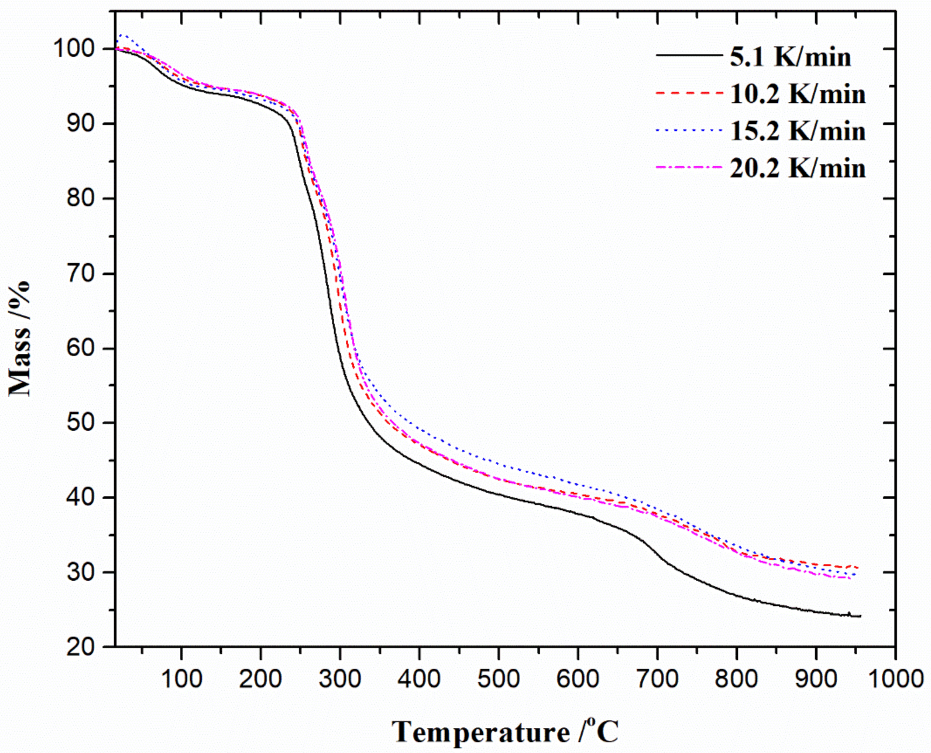

Figure S1. Thermogravimetric curves of non-isothermal thermal decomposition process of UF-HC composite, at the different heating rates (β = 5.1, 10.2, 15.2, and 20.2 K/min).

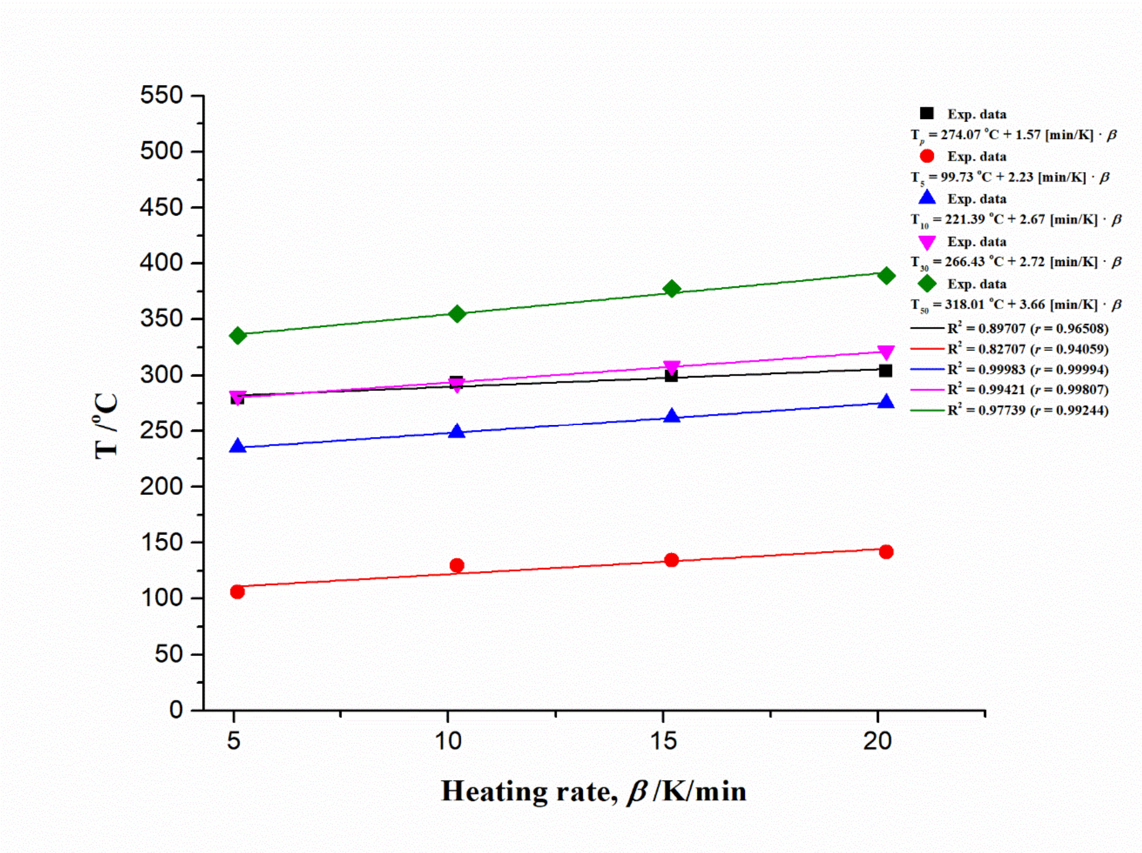

Figure S2. Temperature dependence T_p, T_5, T_{10}, T_{30} and T_{50} on the heating rate (β), for non-isothermal thermal decomposition process of UF-HC composite (the values of linear regression coefficients, are also provided (R^2 and Pearson's (r))).

a)

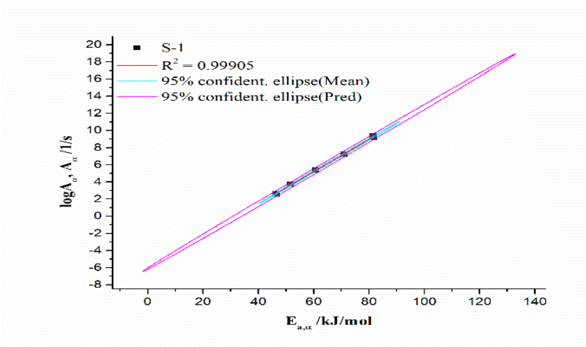

b)

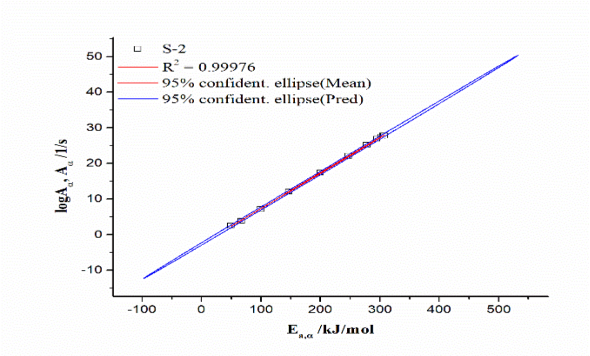

c)

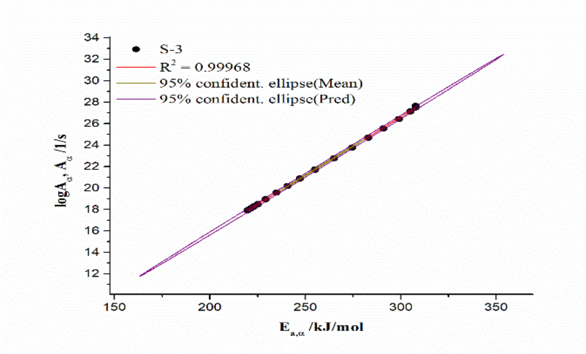

d)

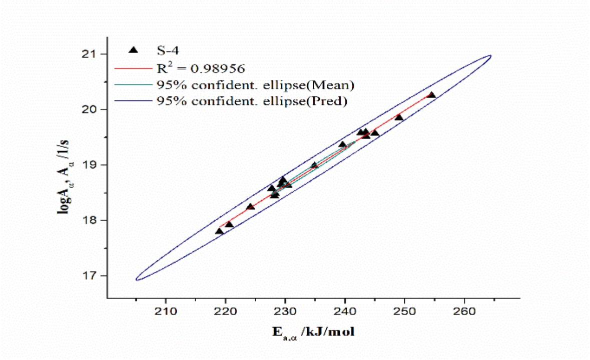

e)

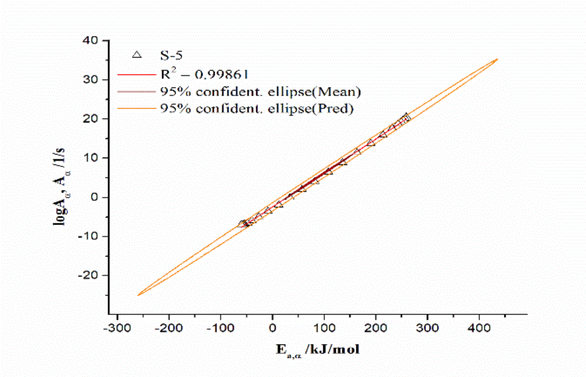

f)

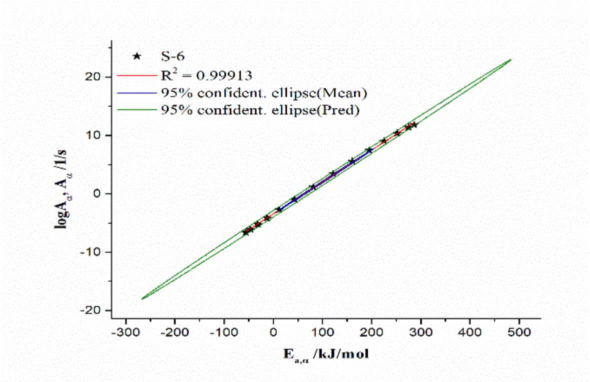

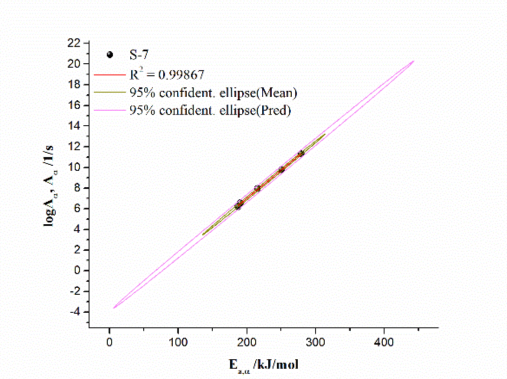

g)

Figure S3. *logAα vs. Ea,α* plots (**a) – g)**) with introduced 95 % confidential ellipses (mean and predicted) regarding to all observed reaction stages (1 – 7), identified from isoconversional kinetic method (NM), and established through seven KCE "branches" (see the main text).

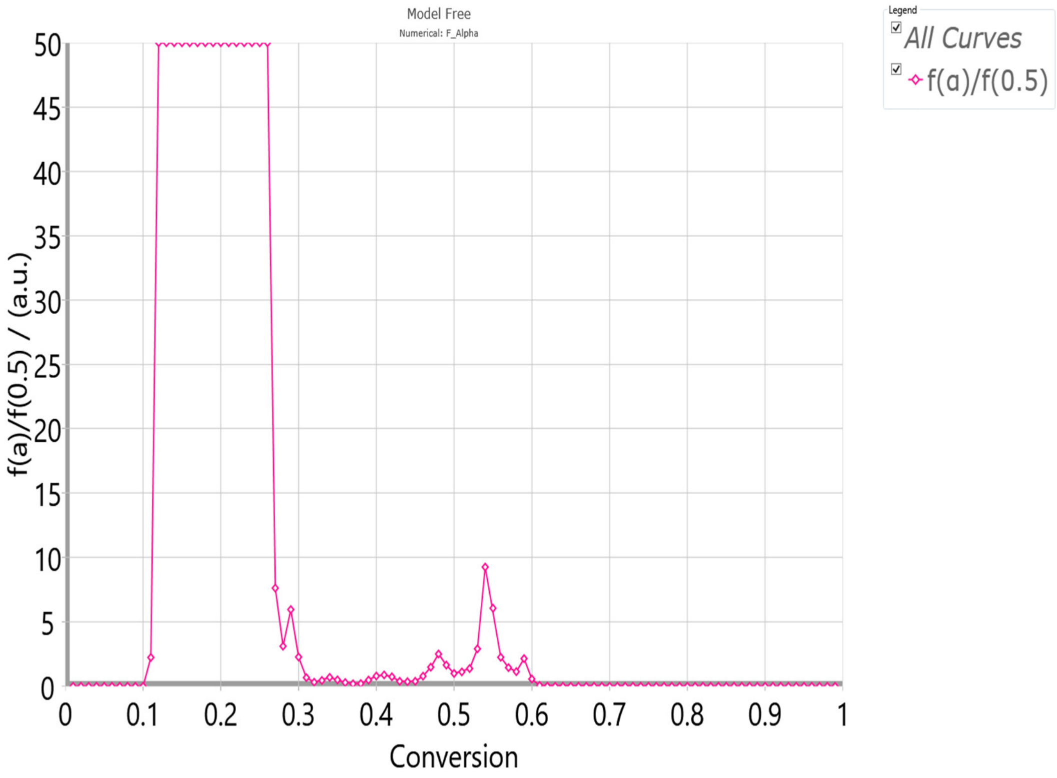

a)

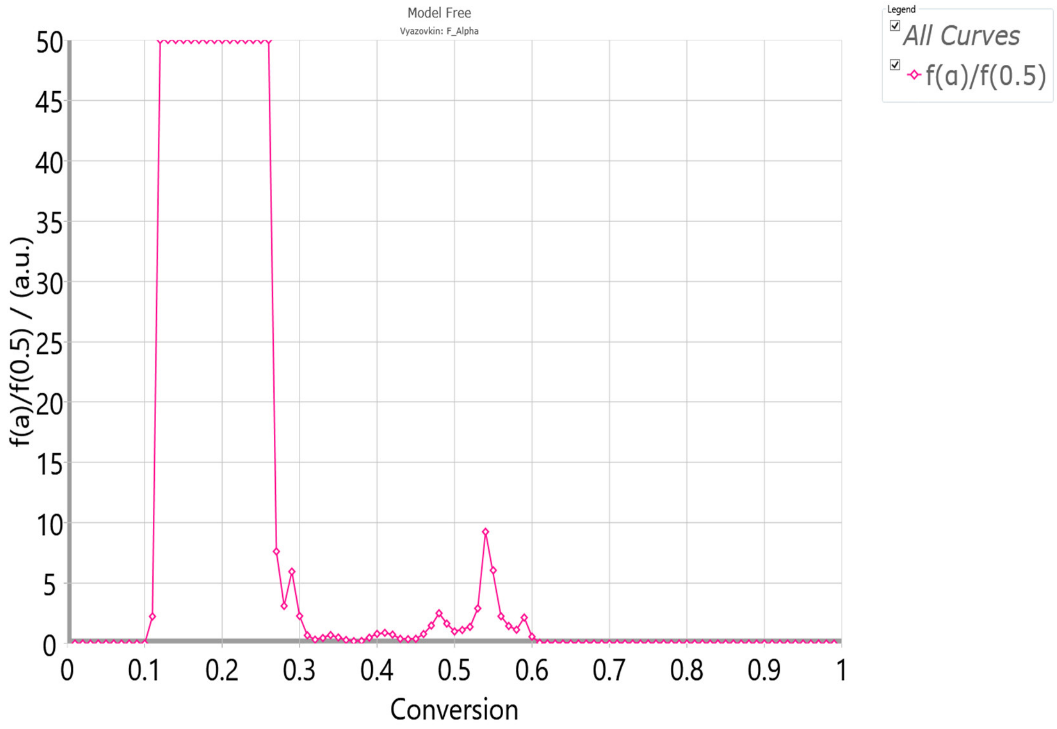

b)

Figure S4. Master plots ($f(\alpha)/f(0.5)$ (a ≡ α) obtained from **a)** numerical optimization (NM) and **b)** Vyazovkin's (VY) model-free (isoconversional) data, for the non-isothermal thermal decomposition process of UF-HC composite.
